# Neutrophil *Irg1*/itaconate axis protects against experimental colitis by suppressing local inflammation and maintaining hematopoietic homeostasis

**DOI:** 10.1186/s43556-025-00390-4

**Published:** 2025-12-19

**Authors:** Na Zhao, Guojian Wang, Shuang Long, Yin Chen, Jining Gao, Xiaofan Lv, Xinze Ran, Yi Jia, Tao Wang

**Affiliations:** 1 Institute of Combined Injury, Chongqing Engineering Research Center for Nanomedicine, School of Preventive Military Medicine, Army Medical University (Third Military Medical University), Chongqing, 400038 China; 2 Institute of Materia Medica and Department of Pharmaceutics, College of Pharmacy, Army Medical University (Third Military Medical University), Chongqing, 400038 China

**Keywords:** *Irg1*, Itaconate, Neutrophil, Inflammatory bowel disease, Experimental colitis, Hematopoietic homeostasis

## Abstract

Inflammatory bowel disease (IBD) is a chronic, relapsing disorder characterized by excessive inflammation and often associated with extraintestinal symptoms. Current treatments remain unsatisfactory. Although *immune response gene 1* (*Irg1*) and its product itaconate show promise in alleviating experimental colitis, the underlying mechanisms are unclear. Here, we describe an endogenous, homeostatic pattern that controls both local and systemic inflammatory responses in experimental murine colitis. Our study identifies neutrophils in inflamed colon as the primary *Irg1* source. *Irg1* deficiency worsens disease severity, as shown by greater weight loss, higher disease activity index, shorter colon length, more severe tissue damage, and more neutrophil infiltration. Depleting neutrophils with Ly6G antibody worsened symptoms in wild-type mice but improved them in knockout mice, highlighting the key role of the *Irg1*/itaconate axis in neutrophil-mediated protection. Blood analysis showed that *Irg1* deficiency increased inflammatory cells and worsened anemia. Bone marrow analysis revealed fewer granulocyte-monocyte progenitors (GMP) and more megakaryocyte-erythroid progenitors (MEP), suggesting a compensatory mechanism. We also found that *Irg1* deficiency increased reverse migrated (rM-ed) neutrophils in blood and bone marrow. Exogenous itaconate (4-octyl itaconate, 4-OI) treatment significantly reduced colon inflammation, lowered rM-ed neutrophil levels, and restored hematopoietic homeostasis. RNA sequencing showed that 4-OI mainly acted by blocking NF-κB signaling, inhibiting endocytosis-related genes, and suppressing rM-ed neutrophils-related genes expression. In conclusion, the neutrophil-derived *Irg1*/itaconate axis plays a key role in mucosal repair and may help maintain hematopoietic balance by regulating rM-ed neutrophils, which suggest that exogenous itaconate derivatives like 4-OI may be effective treatments for IBD.

**Supplementary Information:**

The online version contains supplementary material available at 10.1186/s43556-025-00390-4.

## Introduction

The two major clinically defined forms of inflammatory bowel disease (IBD), Crohn's disease (CD) and ulcerative colitis (UC), are characterized by chronic, relapsing inflammation predominantly affecting the gastrointestinal tract [1–3]. The primary manifestations of IBD encompass abdominal pain, diarrhea and weight loss, which seriously affect the quality of life of patients. The pathogenesis of IBD is intricately intertwined with genetic susceptibility and environmental risk factors, the precise etiology remains elusive. Persistent and uncontrolled abnormal intestinal immune responses are an important feature of IBD, manifested by the aggregation of activated immune cells in the inflamed mucosa, leading to intestinal barrier dysfunction and ulcer formation, which further exacerbates inflammation for intestinal flora exposure [1–3].

Beyond gastrointestinal symptoms, chronic and persistent IBD can cause systemic effects, presenting with extraintestinal manifestations, such as anemia, skin lesions, osteoporosis, and fatty liver, among others [4, 5]. Although these manifestations vary across tissues, they are thought to stem from immune dysregulation and subsequent overactivation. As immune cells originate from the hematopoietic system, inflammation can profoundly affect hematopoietic stem and progenitor cells (HSPCs) [6–8]. Thus, hematopoietic changes in IBD are closely related to local and systemic inflammatory responses. However, the dynamic effects of IBD-related inflammation on HSPCs remains poorly understood.

Immune cells form a complex regulatory network that drives inflammation in IBD [1–3]. Growing evidence highlights neutrophils as key players in this process [9, 10]. During the active phase of IBD, neutrophils accumulate in the affected areas in large numbers and exacerbate the inflammatory response through the secretion of pro-inflammatory cytokines, activation of the NLRP3 inflammasome, and formation of extracellular traps. However, studies in murine colitis models show that neutrophil depletion worsens tissue damage and impaired epithelial repair, suggesting a protective role of neutrophil-derived IL-22 [11]. Research involving clinical patients with IBD further reveals that the CD177^+^ neutrophil subpopulation promoted intestinal epithelial repair by producing IL-22 and TGF-β [12]. These results indicate that neutrophils have dual functions of "destruction" and "protection", but how they switch and integrate in colonic injury and how they participate in the occurrence and development of IBD deserve further study. Meanwhile, emerging evidence indicates that neutrophils are capable of reverse migration from the inflammatory site back into the vasculature following initial infiltration, contributing to the resolution of local inflammatory response or dissemination of inflammation [13, 14]. The precise role and significance of these reverse migrated neutrophils in the pathogenesis and progression of IBD remain to be elucidated.

Recently, the role of itaconate in IBD has attracted increasing attention. Itaconate is a metabolic derivative generated by the host *immune response gene 1* (*Irg1*, also referred to as aconitate decarboxylase 1, ACOD1) during the catalysis of cis-aconitate in the mitochondrial tricarboxylic acid (TCA) cycle [15–17]. A large number of studies have shown that itaconic acid can exert anti-inflammatory effects through mechanisms such as activating the NRF2 antioxidant signaling pathway, inhibiting pyroptosis, and regulating metabolic reprogramming, thereby serving as a significant endogenous protective factor of the organism [15, 16]. Earlier studies showed that the *Irg1*/itaconic acid pathway mainly functions in myeloid cells, especially classically activated macrophages, but the role of neutrophil-derived itaconate has gradually been revealed [18–21]. Recent studies have shown that itaconate plays a protective role in experiment colitis [22–24]. However, the exact cellular source of itaconate in colitis remains unknown, highlighting the need for further investigation into its underlying mechanism of action.

In this study, we demonstrated that *Irg1* was significantly upregulated in mature and activated neutrophils in the affected areas of DSS-induced acute colitis. *Irg1* deficiency exacerbated experimental colonic injury, promoted excessive inflammatory responses, and increased levels of pro-inflammatory cytokines/chemokines. After neutrophil depletion in the experimental colitis model, wild-type (WT) mice showed aggravated tissue damage, while *Irg1* KO mice showed a certain degree of protective effect. Notably, *Irg1* deficiency not only exacerbated systemic inflammatory cell elevation and anemia phenotypes but also disrupted hematopoietic stem cell differentiation in the bone marrow. Consistent with these findings, the numbers of reverse migrated (rM-ed) neutrophils in both the peripheral blood and bone marrow were significantly elevated in KO mice. Furthermore, exogenous 4-OI treatment effectively alleviated local colitis-associated inflammation and restored hematopoietic homeostasis. In vitro RNA-Seq analysis of neutrophil models revealed that 4-OI treatment significantly inhibited LPS-induced inflammatory response, endocytosis-related pathways, and reduced the expression of genes associated with rM-ed neutrophils. In conclusion, the results of this study indicate that maintaining the activity of the neutrophil *Irg1*/itaconate axis exerts a beneficial effect on both local inflammation control and systemic hematopoietic homeostasis restoration in colitis, suggesting that itaconate may have a promising clinical translational prospect for IBD-like diseases.

## Results

### Upregulated *Irg1* is predominantly expressed in neutrophils in the murine colitis model

**Fig. 1:**
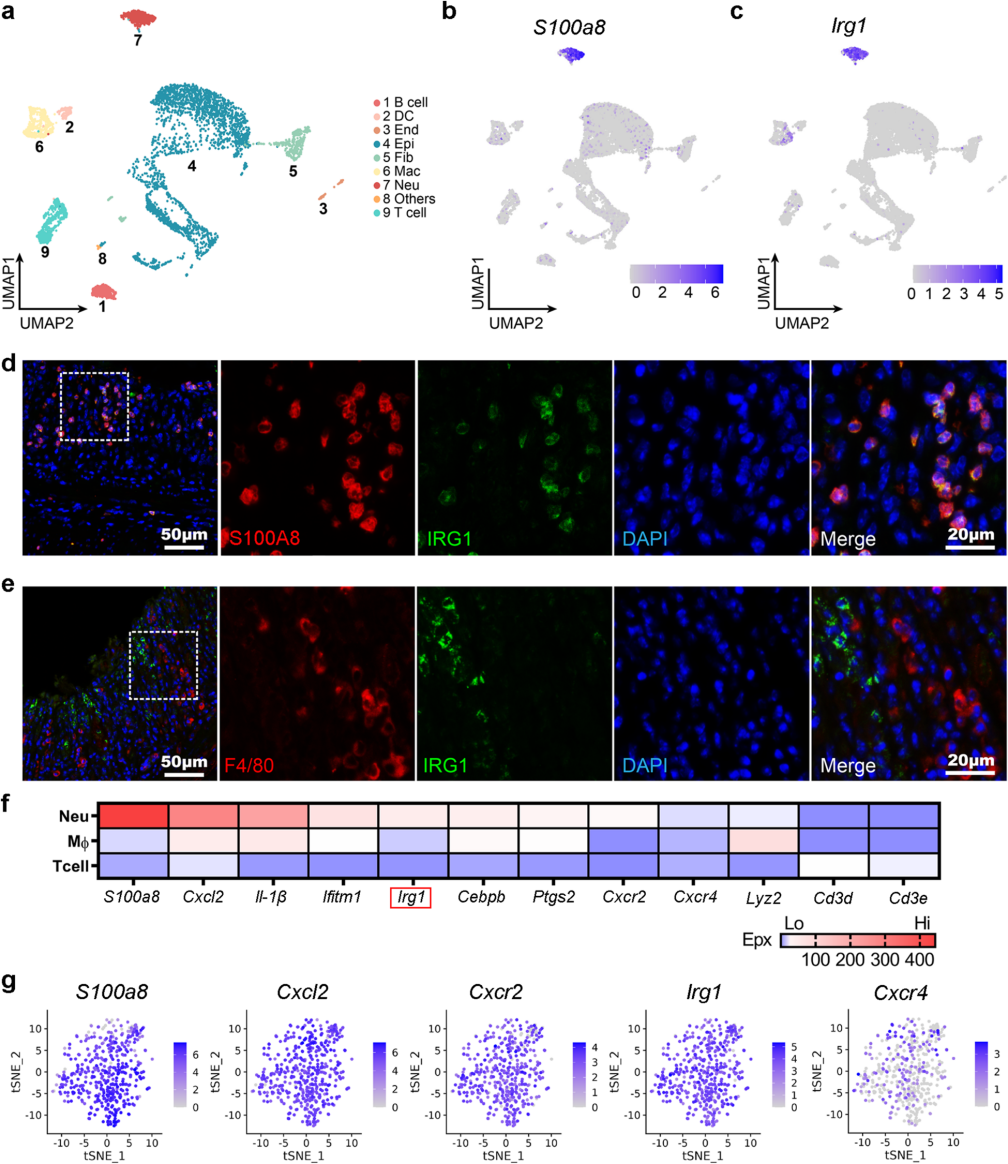
*Irg1* is significantly upregulated in mature neutrophils in the murine colitis model. **a** UMAP visualization of scRNA-Seq cell type from colon tissue samples on the 7th day in 3.0% DSS-induced colitis (n = 5,663 cells). **b** UMAP feature plot of the neutrophil marker *S100a8*. **c** UMAP feature plot of *Irg1* expression. **d** Double IF staining for IRG1(green) and Ly6G (red) in colitis tissue. **e** Double IF staining for IRG1(green) and F4/80 (red) in colitis tissue. **f** Heatmap analysis showing select genes expression associated with the inflammatory response within neutrophils, macrophages and T cells. **g** t-SNE visualization of feature plot of *S100a8*, *Cxcl2*, *Cxcr2*, *Irg1* and *Cxcr4* in the neutrophils from colitis

The *Irg1*/itaconate axis has exhibited a significant protective effect in animal models of inflammatory bowel disease (IBD) [22–24]. However, the cellular source of *Irg1* in colitis models remains poorly studied. To explore the expression pattern of *Irg1*, we established an experimental colitis model by feeding mice with drinking water containing 3% dextran sulfate sodium (DSS) and performed 10 × single-cell RNA sequencing and analysis on colon tissue samples on the 7th day of modeling. The results showed that there was a significant increase in the infiltration of immune cells such as neutrophils, macrophages, T cells, and B cells in the colitis tissue samples (Fig. 1a and Fig. S1), which is consistent with previous reports [25]. Further analysis of the cell types expressing *Irg1* revealed that *Irg1* was predominantly highly expressed in neutrophils, with a minor degree of moderate expression observed in macrophages (Fig. 1b-c). To validate these results, we employed double immunofluorescence (IF) labeling of IRG1 and cell-marker molecules of neutrophils and macrophages. The findings indicated that IRG1 displayed distinct co-localization signals with S100A8-positive neutrophils (Fig. 1d and Fig. S2). In contrast, only a minimal degree of co-localization was observed with F4/80-positive macrophages (Fig. 1e). Further heatmap analysis of select genes associated with the inflammatory response indicated that *Irg1*, along with *Cxcl2*, *Il-1β*, *Cxcr2*, etc., were relatively selectively upregulated in neutrophils (Fig. 1f). The tSNE analysis of the neutrophil population showed that *Irg1* and genes closely related to mature activation such as *S100a8*, *Cxcl2*, and *Cxcr2* were highly expressed in a relatively uniform pattern, while the *Cxcr4* gene related to aging was highly expressed only in a small number of neutrophils (Fig. 1g). These results indicate that *Irg1* is mainly induced and upregulated in mature activated neutrophils at the inflammatory injury sites of murine acute colitis.

### *Irg1* deficiency aggravates murine colitis with enhanced neutrophils infiltration

**Fig. 2:**
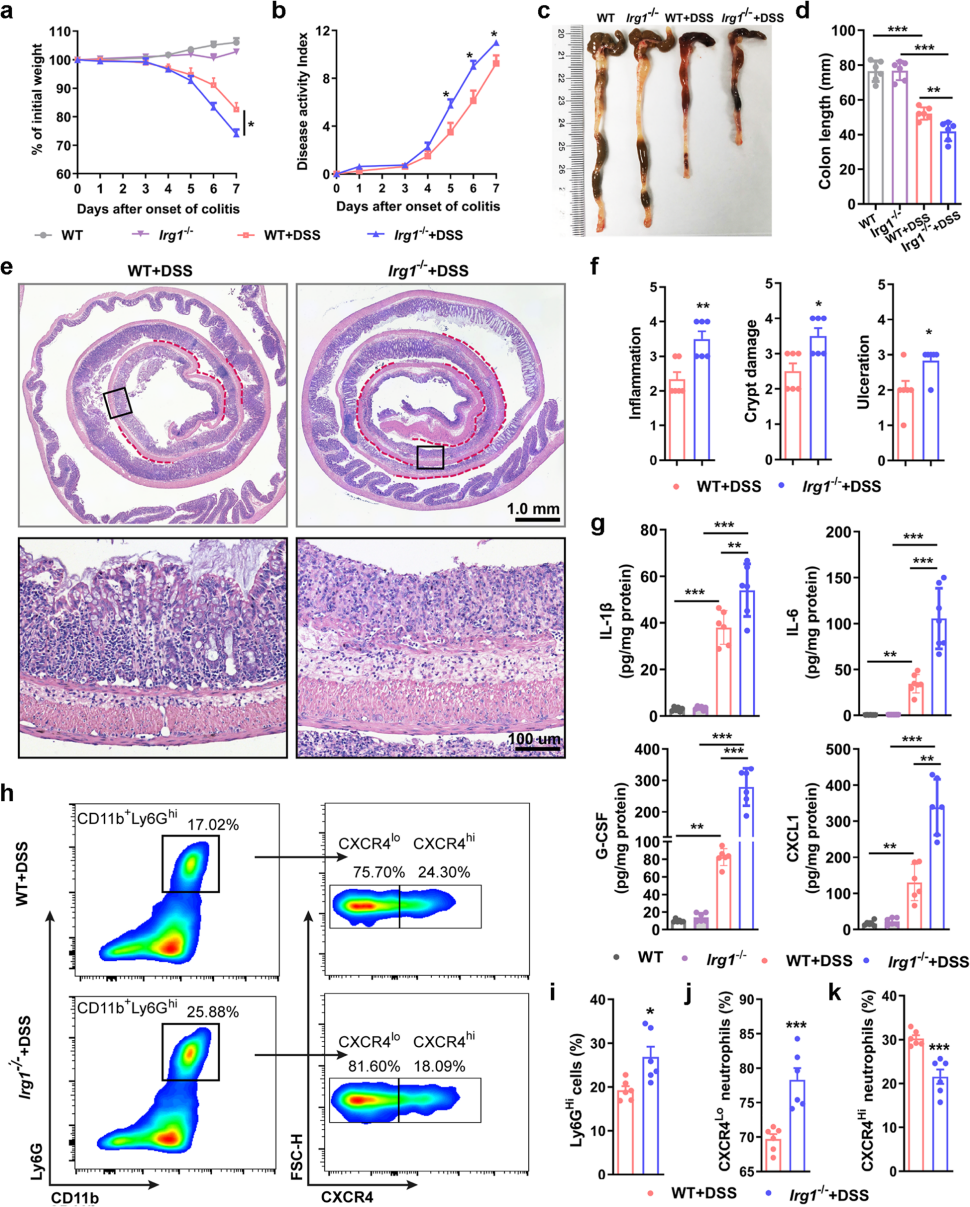
Loss of *Irg1* exacerbates colitis with enhanced neutrophils infiltration in 7-day-DSS colitis model. **a** and **b** Body weight loss (**a**) and DAI scores (**b**) in WT and *Irg1*^−/−^ mice treated with 3.0% DSS ad libitum for consecutive 7 days (n = 8 mice/group). **c** Representative pictures of colons on day 7 after DSS induction. **d **Colon length quantification on day 7 after DSS treatment (n = 8 mice/group). **e** Representative H&E staining colonic Swiss rolls and their partial enlarged views. Red line labelled the ulceration area. **f** Histological severity scores of colonic inflammation, ulceration, and crypt damage were assessed on day 7 after DSS treatment (n = 6 samples/group). **g** Inflammatory cytokines (IL-1β, IL-6, G-CSF and CXCL1) in colon homogenates examined with ELISA (n = 6 samples/group). **h** Representative flow cytometry plots of neutrophil subtypes from the colon of colitis mice at day 7 after DSS treatment. **i** The percentage of neutrophils in colon (n = 6 samples/group). **j** and **k** Frequencies of CXCR4^lo^ (**j**) and CXCR4^hi^ (**k**) neutrophil subsets in colonic neutrophils (n = 6 samples/group). Data are represented as the mean ± SD. ^*∗*^* p* < 0.05, ^*∗∗*^* p* < 0.01, ^*∗∗∗*^* p* < 0.001 versus DSS-treated WT group. Data are representative of three independent experiments

Previous studies have demonstrated that *Irg1*/itaconate axis has a significant protective effect on experimental colitis [22, 23]. Our results further corroborate this conclusion. The *Irg1* mRNA expression levels as well as IRG1 protein levels in the colitis tissue were significantly increased, and the KO mouse exhibited an ideal knockout effect (Fig. S3a-b). As shown in Fig. 2a and b, compared with WT littermate control mice, *Irg1* KO mice showed more significant weight loss and higher disease activity index (DAI) scores during colitis induction. On the 7th day of DSS exposure, the colon length of KO mice was significantly shorter, indicating more severe colonic damage (Fig. 2c-d). Further histopathological images revealed that the ulcer area in the colon of KO mice was wider, and there was a large infiltration of inflammatory cells in both the mucosal and submucosal layers (Fig. 2e). Pathological scoring further confirmed that the scores for ulceration, crypt loss, and inflammatory cell infiltration were significantly higher in *Irg1*-deficient mice (Fig. 2f). Further ELISA (Fig. 2g) and qRT-PCR (Fig. S3a) results showed that the protein and mRNA levels of inflammatory factors IL1-β, IL6, G-CSF and CXCL1 in the colon tissue homogenates of KO colitis mice were significantly higher than those of WT mice. Given that *Irg1* is predominantly expressed in neutrophils within colitis tissues, we further investigated the effect of its deficiency on the number and subtypes of neutrophils in the damaged area. IF staining combined with semi-quantitative statistical analysis showed that the number of S100A8-positive neutrophils in the damaged colon tissue of KO colitis mice increased more significantly (Fig. S3c). Flow cytometry analysis indicated that the relative abundance of CD11b^+^Ly6G^hi^ neutrophils in the colitis tissue of KO mice was significantly higher than that in WT group (Fig. 2h-i and Fig. S3d). Further addition of the CXCR4 surface marker to distinguish mature activated neutrophil subtypes (CXCR4^lo^) and senescent neutrophil subtypes (CXCR4^hi^) revealed that the relative abundance of the mature activated neutrophil subtype Ly6G^hi^ CXCR4^lo^ in the colon of KO mice increased significantly (Fig. 2j). Meanwhile, the relative abundance of the senescent neutrophil subtype Ly6G^hi^ CXCR4^hi^ was significantly lower in the KO group than in the WT group (Fig. 2k). Collectively, these results suggest that *Irg1* deficiency aggravates experimental colitis injury in mice and increases mature activated neutrophils in the inflamed colon tissue.

### Neutrophil depletion worsens colitis in WT mice but reduces inflammation in *Irg1* KO mice

**Fig. 3:**
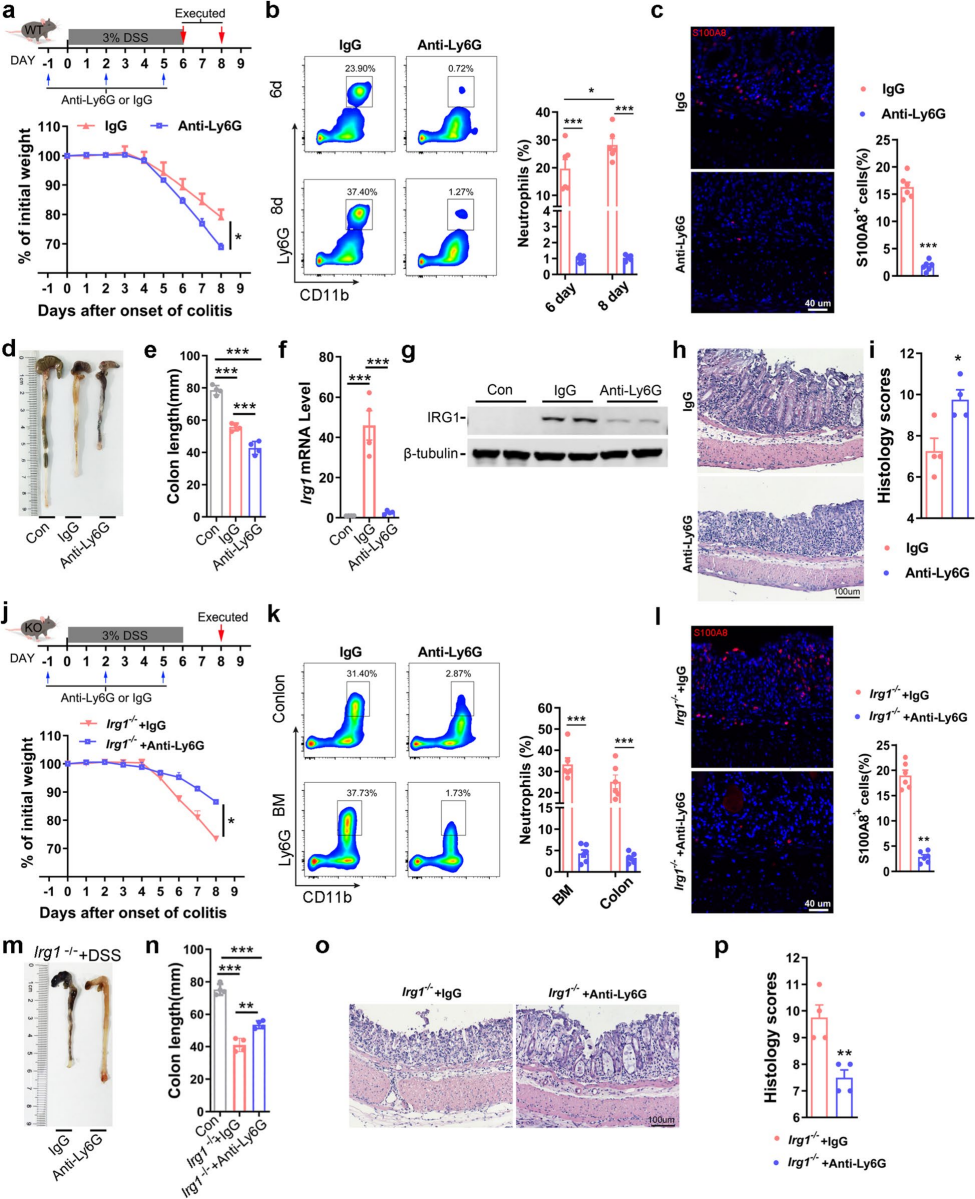
Neutrophil depletion worsens colitis in WT mice but reduces inflammation in Irg1 KO mice. **a** Schematic of neutrophil depletion in WT mice and quantitation of body weight loss after DSS induction (n = 5 mice/group). **b **Flow cytometry: Percentage of Ly6G⁺CD11b⁺neutrophils in total colonic leukocytes at day 6 and day 8 after DSS treatment (n = 6 samples/group). **c** Representative S100A8 IF staining (red) in colon sections of WT mice (left) and quantification of S100A8 positive neutrophils among nucleated cells(right). **d** and** e** Representative pictures of colons (**d**) and length quantification (**e**) on day 8 after DSS treatment in WT mice (n = 5). **f** *Irg1* mRNA expression (RT-qPCR) in colon tissues on day 8 after DSS induction (n = 4 samples/group). **g** IRG1 protein expression (western blot) on day 8 after DSS induction (n = 4).** h** Representative H&E-stained colon images on day 8 after DSS induction in WT mice. **i** Histological scores for colitis severity (including crypt architecture, immune infiltration, and ulceration) were assessed on day 8 after DSS treatment in WT mice (n = 4). **j** Schematic of neutrophil depletion in *Irg1*^*−/−*^ mice and quantitation of body weight loss (n = 5 mice/group). **k** Flow cytometry: Percentage of Ly6G⁺CD11b⁺neutrophils in total bone marrow and colonic leukocytes on day 8 after DSS treatment in *Irg1*^*−/−*^mice. **l** Representative S100A8 IF staining (red) in colon sections of *Irg1*^*−/−*^mice (left) and quantification of S100A8 positive neutrophils among nucleated cells(right). **m** and **n** Representative pictures of colons (**m**) and length quantification (**n**) on day 8 after DSS treatment in *Irg1*^*−/−*^mice (n = 5). **o** Representative H&E-stained colon images on day 8 after DSS treatment in *Irg1*^*−/−*^mice. **p** Histological scores for colitis severity (including crypt architecture, immune infiltration, and ulceration) were assessed on day 8 after DSS treatment in *Irg1*^*−/−*^mice (n = 4). Data are represented as the mean ± SD. ^*∗*^* p* < 0.05, ^*∗∗*^* p* < 0.01, ^*∗∗∗*^* p* < 0.001. Data are representative of three independent experiments

To investigate the role of *Irg1*/itaconate axis in neutrophil during colitis development, we depleted neutrophils using an anti-Ly6G antibody and performed a series of experiments. Consistent with previous findings, intraperitoneal administration of anti-Ly6G antibodies significantly reduced neutrophil levels in both peripheral blood and bone marrow of the experimental colitis model (Fig. S4a-f), while simultaneously exacerbating weight loss (Fig. 3a). Further flow cytometry analysis and IF examination of colitis tissues indicated that anti-Ly6G injection did significantly reduce the level of neutrophils in colon (Fig. 3b-c). And neutrophil depletion resulted in more pronounced colon shortening in mice (Fig. 3d-e). Quantitative PCR and Western blot analyses revealed that neutrophil depletion markedly decreased *Irg1* mRNA and IRG1 protein expression levels, thereby corroborating the single-cell sequencing results indicating that Irg1 is predominantly expressed in neutrophils (Fig. 3f-g, and Fig.S4g-h). Pathological evaluations further demonstrated that neutrophil depletion intensified colonic tissue damage (Fig. 3h-i). As previously described, *Irg1* knockout mice in the experimental colitis model exhibited aggravated injury accompanied by increased neutrophil infiltration (Fig. 2). This raises the question: do neutrophils lacking *Irg1* expression retain their protective capacity? To address this, we conducted a neutrophil depletion experiment in the colitis model of *Irg1* KO mice. The results indicated that following anti-Ly6G antibodies injections in KO mice, weight loss was attenuated (Fig. 3j), and indeed significantly reduced neutrophil levels in colitis tissues as well as bone marrow (Fig. 3k-l). What’s more, neutrophil depletion in KO mice led to mitigated colon shortening and improved pathological scores (Fig. 3m-p). Collectively, these findings underscore the importance of the *Irg1*/itaconate axis for the protective role of neutrophils in experimental colitis.

### *Irg1* deficiency leads to compensatory differentiation changes in GMP and MEP in colitis mice

**Fig. 4:**
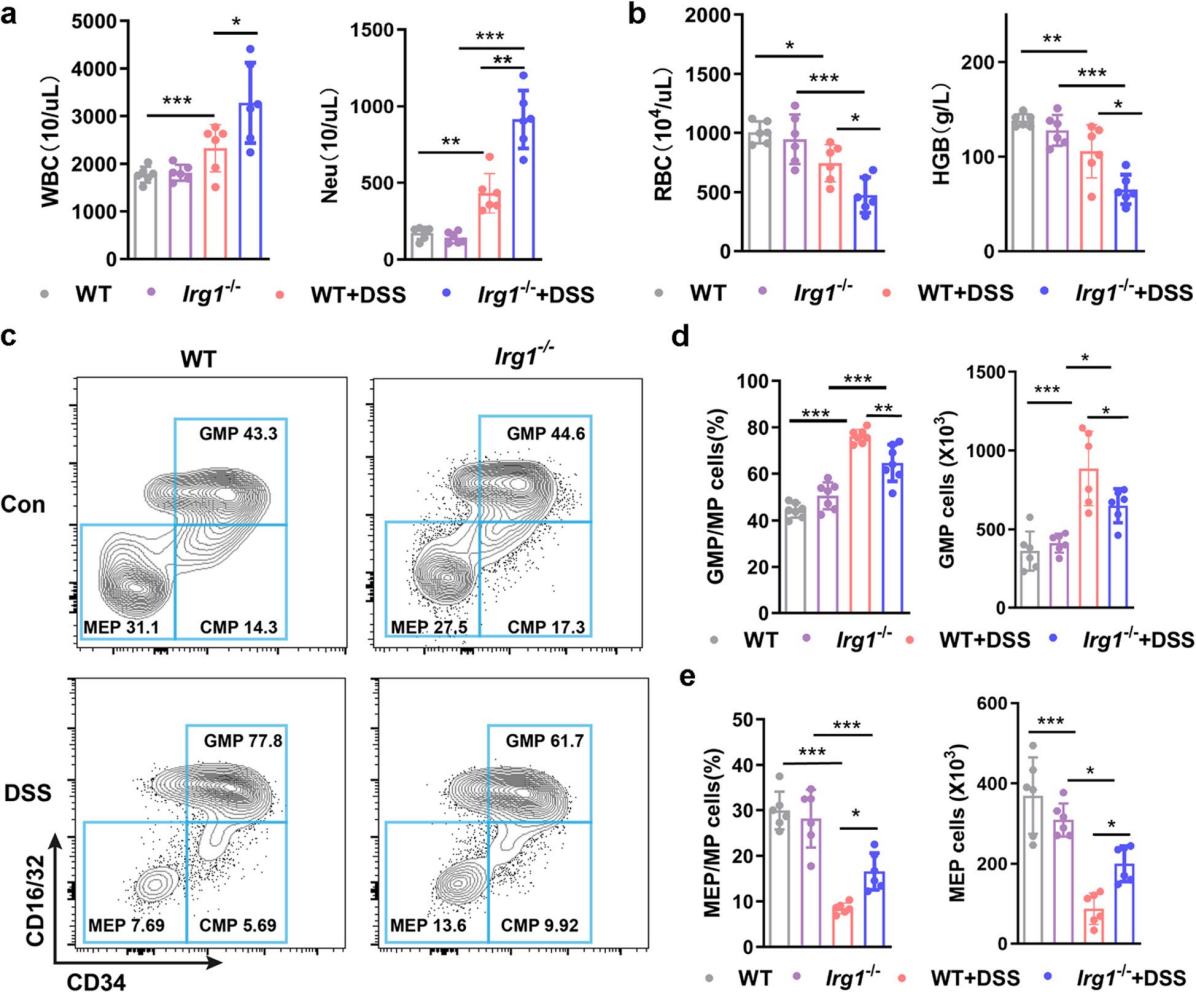
*Irg1* deficiency disrupts GMP and MEP dynamics in DSS-induced colitis. **a** Peripheral blood cell counts for white blood cells (WBCs, left) and neutrophils (right) in WT and *Irg1*^−/−^ mice on day 7 after 3.0% DSS exposure (n = 6 samples/group). **b** Peripheral blood counts for red blood cells (RBCs, left) and hemoglobin (HGB, right) on day 7 after DSS induction (n = 6 samples/group). **c** Flow cytometry profiles of bone marrow GMP, MEP, and CMP in untreated and DSS-treated WT and *Irg1*^−/−^ mice on day 7 after DSS exposure. **d** and **e** Frequency and absolute numbers of GMP (**d**) and MEP (**e**) in bone marrow (single-cell suspensions from one femur and tibia) on day 7 after DSS treatment (n = 6 samples/group). Data represent mean ± SD. *P* values: ^*∗*^* p* < 0.05, ^*∗∗*^* p* < 0.01, ^*∗∗∗*^* p* < 0.001. Results are representative of three independent experiments

Persistent inflammatory responses often lead to emergency hematopoiesis in bone marrow characterized by myeloid-biased differentiation, and Irg1 may be involved in this process [6–8]. We hypothesized that the maintenance of the *Irg1*/itaconate axis activity in neutrophils would have impact on emergency hematopoiesis of bone marrow in experimental colitis. Peripheral blood WBC counts results showed that *Irg1* deficiency further exacerbated the elevated levels of WBC and neutrophils in experimental colitis mice (Fig. 4a), while having little effect on the numbers of monocytes and lymphocytes (Fig. S5a). Detection of peripheral blood RBC counts and hemoglobin content revealed that *Irg1* deficiency aggravated anemia in colitis mice (Fig. 4b), but had limited impact on platelet levels (Fig. S5a). Flow cytometry analysis of bone marrow HSPCs showed that DSS-induced experimental colitis led to an emergency increase in LSK hematopoietic stem cells and MP progenitor cells, but there were no differences between *Irg1* KO mice and WT mice (Fig. S5b-c). Further analysis revealed that experimental colitis indeed caused GMP-biased differentiation of HSPCs, as evidenced by a significant increase in the proportion and number of GMPs compared to normal control mice, accompanied by a significant decrease in the proportion and number of MEPs (Fig. 4c-e). However, surprisingly, compared with WT colitis mice, the relative abundance and number of GMPs in bone marrow of KO colitis mice were significantly reduced (Fig. 4d), while the proportion and number of MEPs were relatively increased (Fig. 4e). In addition, the absence of *Irg1* had little effect on the proportion and number of common myeloid progenitors (CMPs) (Fig. S5d). This seemingly contradictory result between peripheral blood WBC and RBC counts and bone marrow hematopoietic progenitor cell analysis suggests that *Irg1* deficiency further aggravates the systemic inflammatory cell increase and anemia phenotype, thereby leading to adaptive changes in the compensatory differentiation of hematopoietic progenitor cells.

### *Irg1* deficiency increases rM-ed neutrophils in peripheral blood and bone marrow during experimental colitis

**Fig. 5:**
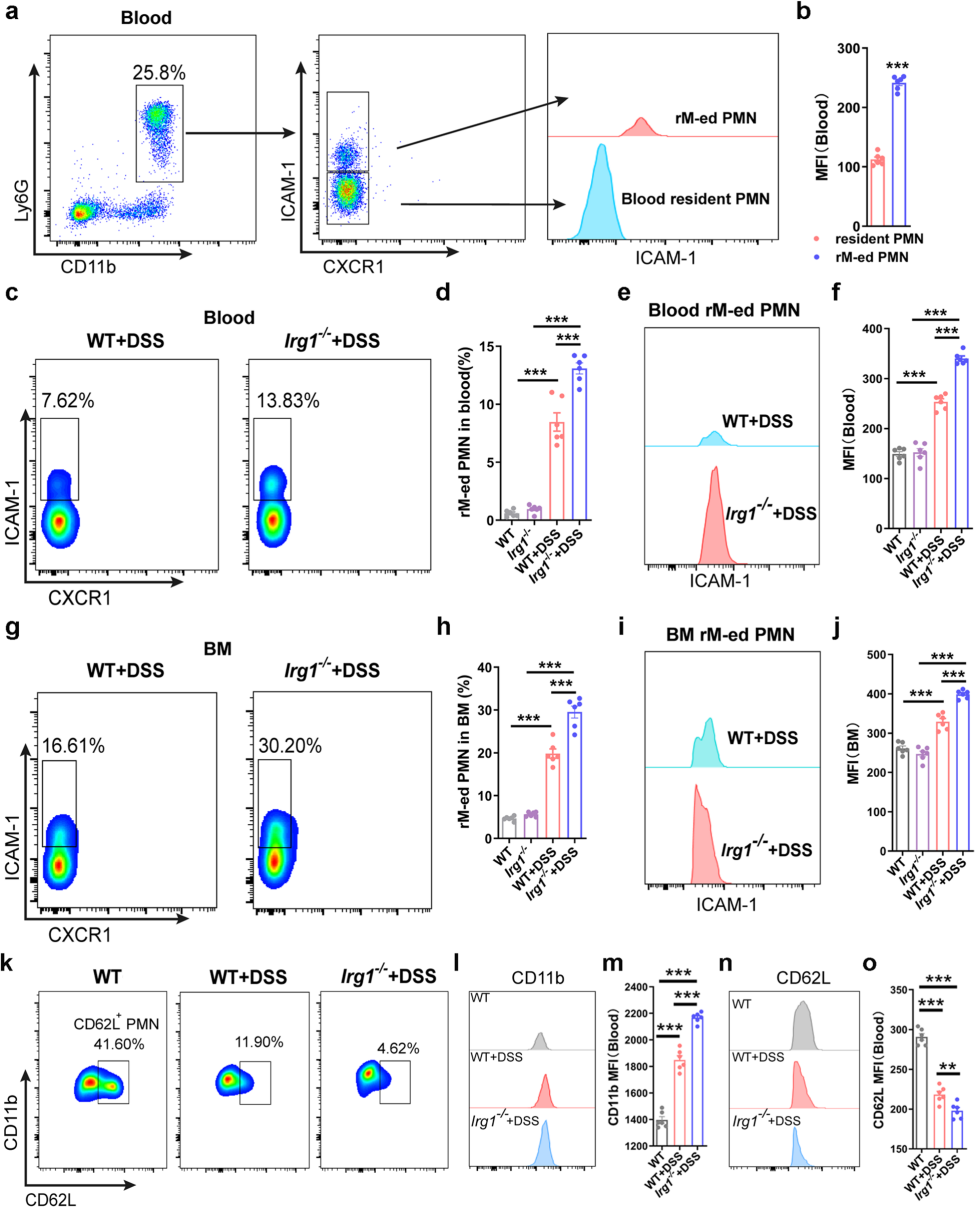
*Irg1* deficiency increases rM-ed neutrophils in peripheral blood and bone marrow during experimental colitis.** a** Representative flow cytometry plots (left) and histograms (right) of rM-ed neutrophils versus tissue-resident neutrophils in peripheral blood of 3.0% DSS-treated WT mice at day 7. **b** Mean fluorescence intensity (MFI) of ICAM-1 on peripheral blood neutrophils (n = 6 samples/group). **c** Representative flow cytometry plots of rM-ed neutrophils in peripheral blood neutrophils of DSS-treated WT and *Irg1*^−/−^ mice. **d** Quantification of rM-ed neutrophils frequency in peripheral blood total neutrophils of control and DSS-treated mice (n = 6 samples/group). **e** and **f** Representative histograms of ICAM-1 expression (**e**) and quantification of ICAM-1 MFI (**f**) on rM-ed neutrophils (n = 6 samples/group). **g** Representative flow cytometry plots of rM-ed neutrophils in bone marrow of DSS-treated WT and *Irg1*^−/−^ mice. **h** Frequency of rM-ed neutrophils in bone marrow total neutrophils of control and DSS-treated mice (n = 6 samples/group). **i** and **j** ICAM-1 expression histograms (**i**) and MFI (**j**) in bone marrow rM-ed neutrophils (n = 6 samples/group). **k** Representative flow cytometry plots of CD62L^+^ neutrophils in peripheral blood neutrophils of DSS-treated WT and *Irg1*^*−/−*^ mice. **l** and **m** CD11b expression histograms (**l**) and MFI (**m**) in peripheral blood neutrophils of control and DSS-treated mice (n = 6 samples/group). **n** and **o** CD62L expression histograms (**n**) and MFI (**o**) in peripheral blood neutrophils of control and DSS-treated mice (n = 6 samples/group). Data represent mean ± SD. *P* values: ^*∗∗*^* p* < 0.01, ^*∗∗∗*^* p* < 0.001. Results are representative of three independent experiments

Recently, neutrophil reverse migration has emerged as a novel regulatory mode of inflammatory responses [13, 14, 26, 27]. Studies have found that *Irg1* upregulates in rM-ed neutrophils [28]. It has also been shown that IRG1-positive neutrophils at tendon injury sites can return to the bone marrow to participate in hematopoietic regulation in response to systemic inflammatory responses [29]. In the context of an experimental colitis model, our findings revealed that *Irg1* knockout exacerbated systemic inflammatory responses and disrupted bone marrow emergency hematopoiesis. We postulate that reverse migrated (rM-ed) neutrophils may be intricately involved in these processes. The rM-ed neutrophils are defined as ICAM-1^hi^CXCR1^lo^, the well-accepted markers of rM-ed neutrophils. In the experimental colitis model, a prominent feature of rM-ed neutrophils was the high expression of ICAM-1, with the mean fluorescence intensity (MFI) level significantly higher than that of resident neutrophils in the blood (Fig. 5a-b and Fig. S6a). Further comparative analysis indicated that the proportion of rM-ed neutrophils in the peripheral blood of *Irg1* KO mice was significantly higher than that of WT mice in the colitis model (Fig. 5c-d). Meanwhile, the MFI of ICAM-1 on rM-ed neutrophils was also significantly elevated in *Irg1* KO mice (Fig. 5e-f). The bone marrow is considered an important destination for rM-ed neutrophils. Flow cytometry analysis revealed a significantly higher proportion of rM-ed neutrophils in the bone marrow of *Irg1* KO mice compared to WT mice (Fig. 5g-h), consistent with peripheral blood findings. Notably, the MFI trend of ICAM-1 on rM-ed neutrophils in the bone marrow mirrored that in peripheral blood, but the expression levels were markedly higher in the bone marrow (Fig. 5i-j). Both rM-ed neutrophils and activated neutrophils exhibit higher expression of CD11b and lower expression of CD62L [30]. Flow cytometry showed that in the colitis model, neutrophils from *Irg1* knockout mice had higher CD11b and lower CD62L MFI values in peripheral blood than those in WT mice, consistent with the rM-ed neutrophil phenotype (Fig. 5k-o). Collectively, these results suggest that *Irg1* deficiency leads to a higher proportion of rM-ed neutrophils in both peripheral blood and bone marrow of colitis model. This finding implies a close association between *Irg1* deficiency and the more severe systemic inflammation and disrupted emergency hematopoiesis phenotypes observed in KO mice.

### 4-Octyl itaconate treatment alleviates experimental colitis

**Fig. 6:**
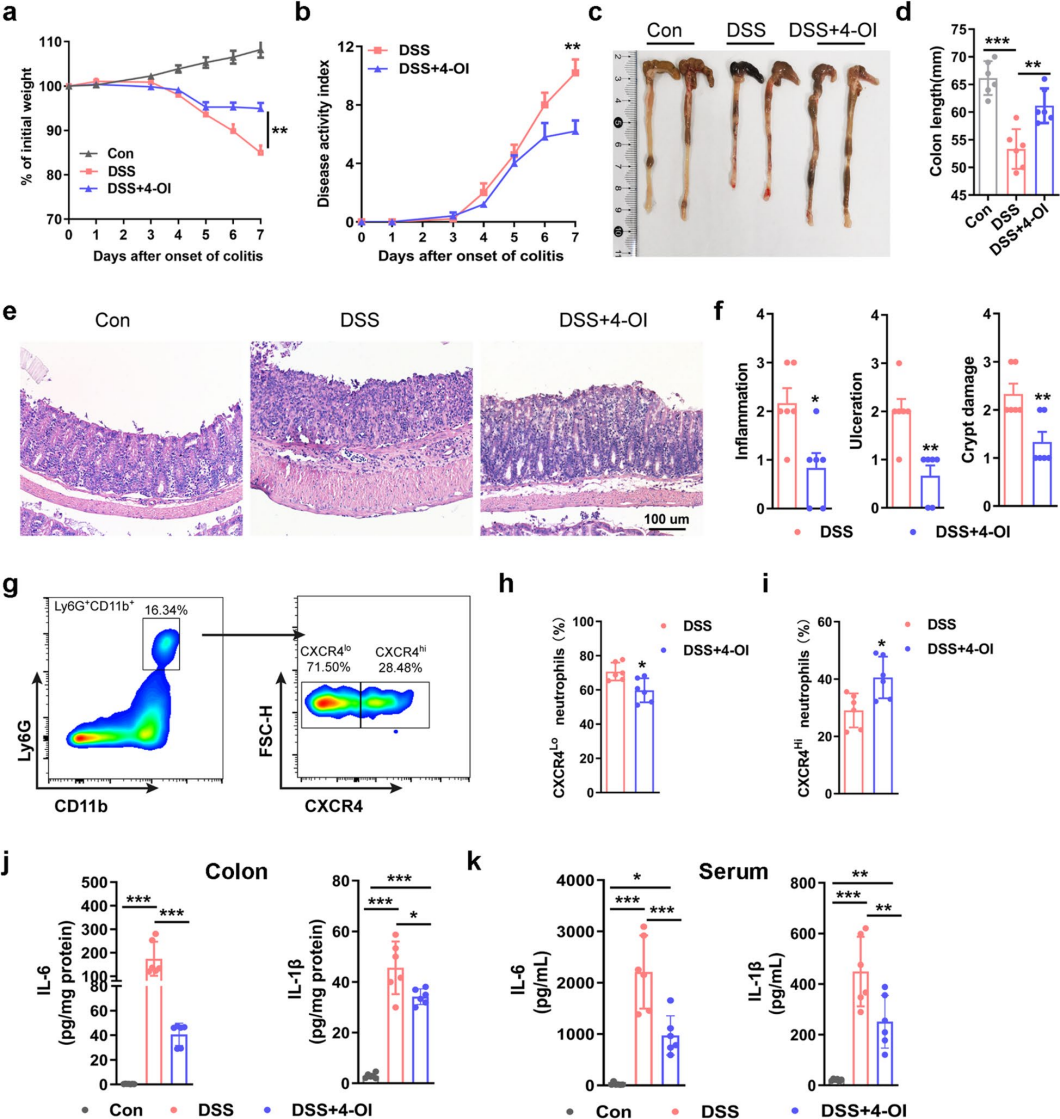
4-OI ameliorates DSS-induced colitis. **a** and **b** Body weight change (**a**) and DAI scores (**b**) in mice administered with 2.5% DSS for 7 consecutive days followed by 4-OI treatment (25 mg/kg, i.p.) or vehicle for 6 days (n = 8 mice/group). **c** Representative pictures of colons on day 7 after DSS induction. **d** Quantification of colon length (n = 6 samples/group). **e** Representative H&E-stained colon images. **f** Histopathological scoring of inflammation severity, ulceration extent, and crypt damage (n = 6 samples/group). **g** Representative flow cytometry profiles of CXCR4^hi^ neutrophil subsets (gated on CD11b⁺Ly6G⁺cells) in colonic lamina propria on day 7 after DSS treatment. **h** and **i** Frequencies of CXCR4^lo^ (**h**) and CXCR4^hi^ (**i**) neutrophil subsets in colon neutrophils (n = 6 samples/group). **j** and **k** Concentrations of both IL-1β and IL-6 in colon tissue homogenates (**j**) and serum (**k**) on day 7 after DSS treatment (n = 6 samples/group). Data are represented as the mean ± SD. ^∗^ *p* < 0.05, ^∗∗^ *p* < 0.01, ^∗∗∗^ *p* < 0.001 versus WT mice group at same time points. Data are representative of three independent experiments

Given the critical protective role of the endogenous neutrophil *Irg1*/itaconate axis in colitis models, we explored exogenous itaconic acid as a therapeutic intervention from a translational standpoint to assess its efficacy in ameliorating the key enteritis-associated phenotypes of interest. 4-Octyl Itaconate, as an itaconic acid derivative with good cell permeability, is a commonly used exogenous therapeutic supplement form of itaconic acid and has shown protective effects in various metabolic inflammatory diseases. We evaluated the therapeutic effect of 4-OI in a moderate experimental colitis model induced by 2.5% DSS. As shown in Fig. 6a and b, on the 6th day of 4-OI treatment group, the weight loss of colitis mice slowed down and the DAI scores tended to stabilize. Colon length measurement showed that the 4-OI treatment group recovered to a length comparable to that of normal mice, markedly improved compared to the solvent control colitis group (Fig. 6c-d). Further histopathological analysis indicated that 4-OI treatment significantly alleviated colitis-associated pathological damage (Fig. 6e-f). Flow cytometry analysis of colitis tissues showed that 4-OI treatment significantly reduced the proportion of mature activated neutrophils (Ly6G^hi^CXCR4^Lo^) and increased senescent neutrophils (Ly6G^hi^CXCR4^hi^) in the colon (Fig. 6g-i). Meanwhile, ELISA analysis showed that 4-OI treatment significantly reduced levels of pro-inflammatory cytokines (IL-1β and IL-6) in both colitis tissues and serum (Fig. 6j-k). These results suggest that exogenous itaconic acid can effectively alleviate the damage caused by experimental colitis through inhibiting neutrophil infiltration and reducing the levels of pro-inflammatory cytokines.

### 4-Octyl itaconate treatment facilitates the restoration of hematopoietic homeostasis in experimental colitis

**Fig. 7:**
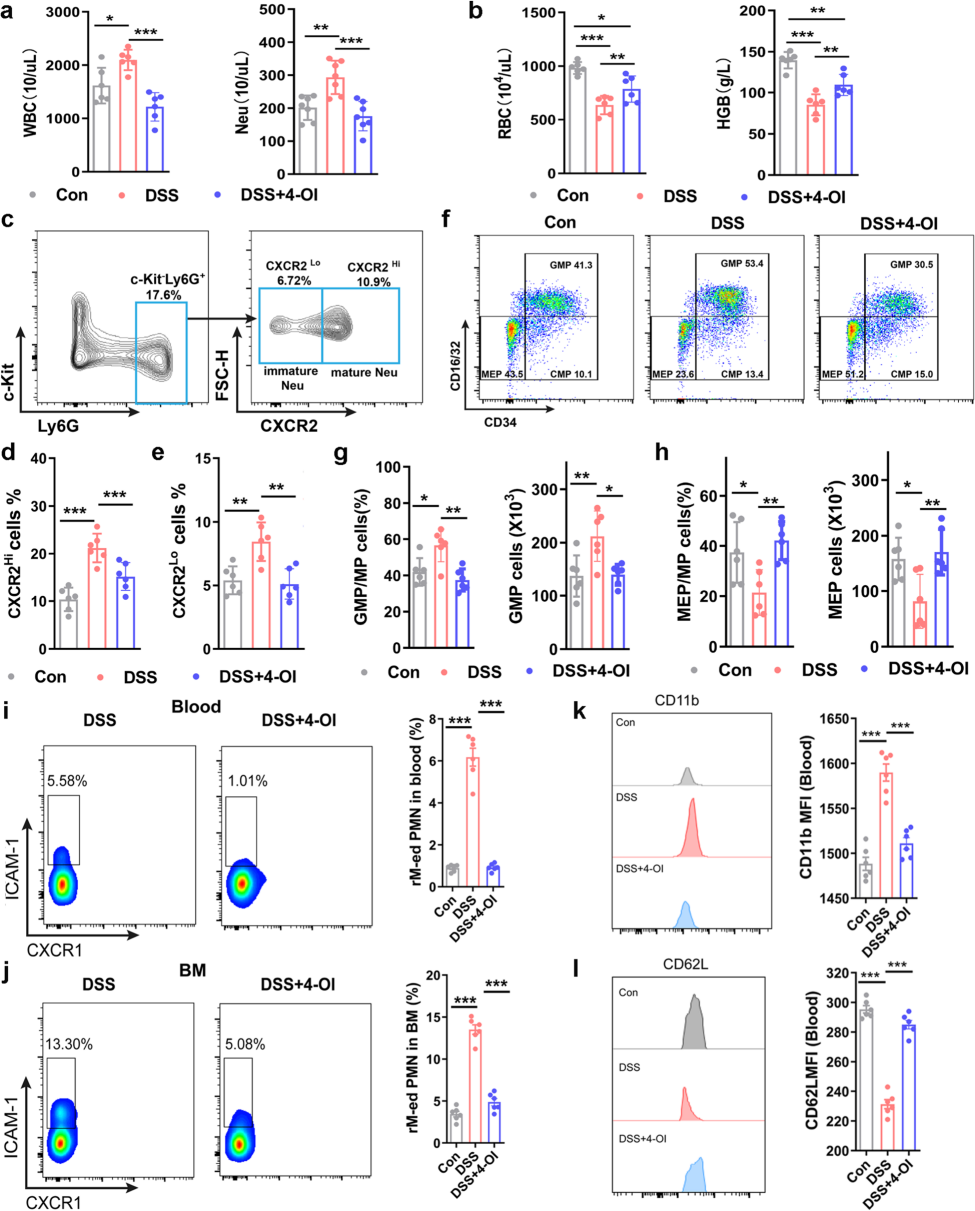
4-OI restores hematopoietic homeostasis in experimental colitis. **a** Peripheral blood counts for WBC (left) and neutrophils (right) in DSS-treated WT mice administered 4-OI (25 mg/kg, i.p.) on day 7 after DSS treatment (n = 8 samples/group). **b** Peripheral blood counts for RBC (left) and HGB (right) levels in peripheral blood (n = 6 samples/group). **c** Representative flow cytometry profiles of bone marrow neutrophil subsets (gated on c-Kit^⁺^Ly6G^⁺^cells) from 4-OI-treated mice. **d** and **e** Frequencies of CXCR2^hi^ (**d**) and CXCR2^lo^ (**e**) neutrophil subsets in bone marrow neutrophil (n = 6 samples/group). **f** Flow cytometry profiles of bone marrow GMP, MEP, and CMP from 4-OI-treated mice on day 7 after DSS treatment (n = 6 samples/group). **g** and **h** Frequency and absolute counts of GMP (**g**) and MEP (**h**) in bone marrow (single-cell suspensions from one femur and tibia) (n = 6 samples/group). **i** Flow plots and quantification of rM-ed neutrophils in peripheral blood neutrophils on day 7 after DSS treatment (n = 6 samples/group). **j** Flow plots and quantification of rM-ed neutrophils in bone marrow neutrophils on day 7 after DSS induction (n = 6 samples/group). **k **CD11b expression histograms and MFI in peripheral blood neutrophils on day 7 after DSS induction (n=6 samples/group). **l **CD62L expression histograms and MFI in peripheral blood neutrophils on day 7 after DSS induction (n=6 samples/group). Data are represented as the mean ± SD. ^∗^ *p* < 0.05, ^∗∗^ *p* < 0.01, ^∗∗∗^ *p* < 0.001 versus WT mice group at same time points. Data are representative of three independent experiments

Subsequently, we evaluated the impact of 4-OI treatment on the elevated levels of circulating inflammatory cells and anemia caused by continuous inflammatory stimulation. As shown in Fig. 7a, 4-OI treatment normalized the significantly elevated WBC and neutrophil counts in peripheral blood of mice with colitis. Meanwhile, 4-OI treatment significantly alleviated the anemia phenotype, characterized by increased RBC count and hemoglobin content in the colitis model (Fig. 7b). Flow cytometry analysis of bone marrow neutrophils revealed that 4-OI treatment significantly reduced the proportion of both mature CXCR2^hi^ and immature CXCR2^lo^ neutrophils in the bone marrow compared to the DSS treatment group (Fig. 7c-e and Fig. S7). Further flow cytometry analysis of bone marrow hematopoietic progenitor cells demonstrated that 4-OI treatment significantly reversed colitis-induced myeloid-biased differentiation, as evidenced by the restoration of GMP proportion and number to normal levels (Fig. 7f-g). Moreover, 4-OI treatment completely alleviated the suppression of MEP differentiation in colitis (Fig. 7h). Given the potential role of rM-ed neutrophils in bone marrow emergency hematopoiesis, we investigated the impact of 4-OI treatment on the levels of rM-ed neutrophils in peripheral blood and bone marrow. The results demonstrated that 4-OI treatment significantly reduced the levels of rM-ed neutrophils in both compartments of colitis mice, restoring them to levels comparable to those in the normal state (Fig. 7i-j). Consistent with the rM-ed neutrophil findings, flow cytometry showed that 4-OI treatment significantly normalized the activated neutrophil phenotype in the peripheral blood of colitis mice, as shown by reduced CD11b expression and increased CD62L expression levels comparable to those in normal control neutrophils (Fig. 7k-l).

### RNA-Seq revealed that 4-OI suppresses LPS-induced neutrophil inflammatory responses by regulating multiple pathways

**Fig. 8:**
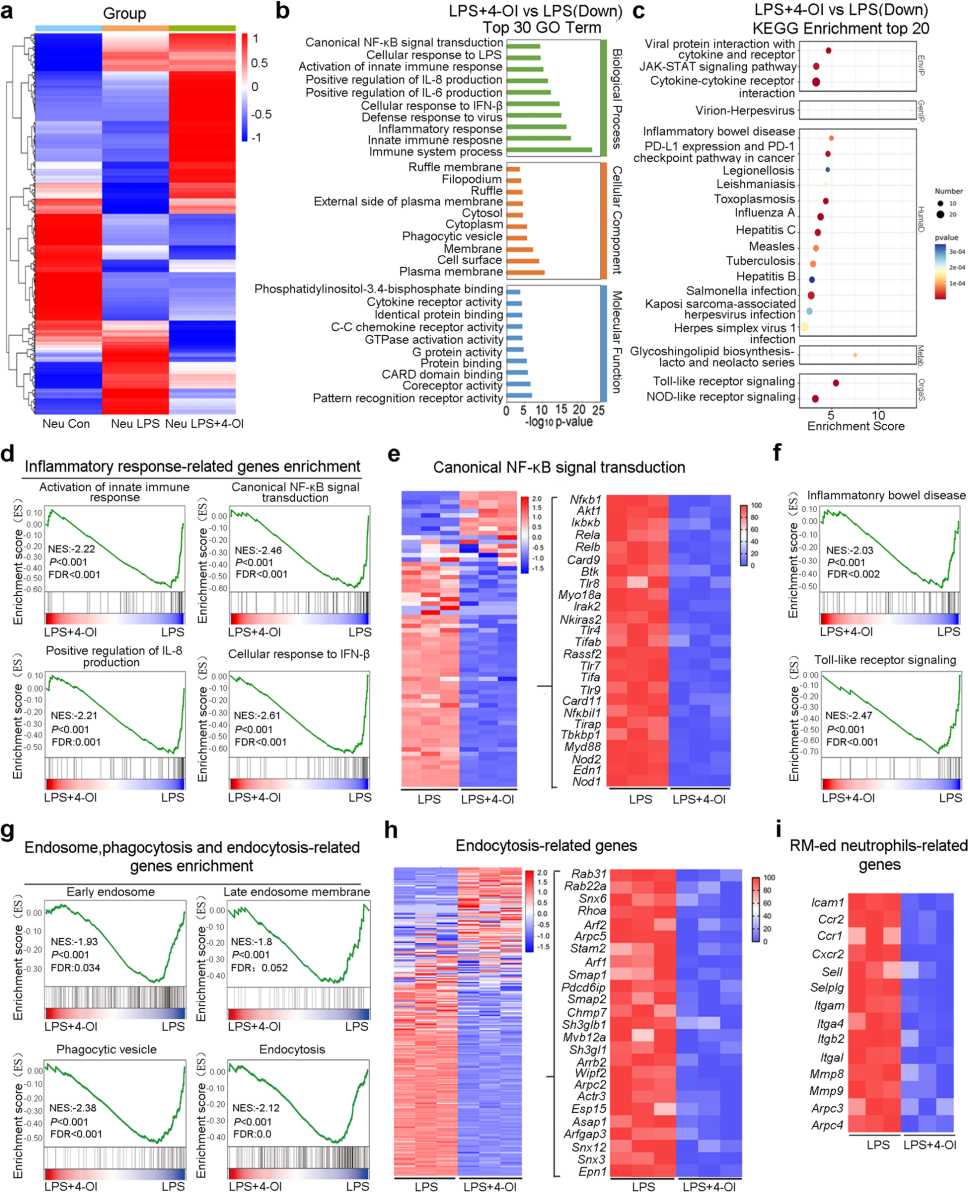
Transcriptomic profiling reveals multi-pathway anti-inflammatory mechanisms of 4-OI on LPS-stimulated neutrophils. **a** Heatmap of differentially expressed genes (DEGs) in 4-OI-treated versus untreated neutrophils (fold change ≥ 2.0, *P* < 0.05). **b** Top 30 significantly enriched GO terms for downregulated DEGs in LPS + 4-OI versus LPS-only groups. **c **Top 20 KEGG pathways enriched in downregulated DEGs. **d** GSEA showing marked downregulation of inflammation-related pathways in 4-OI-treated neutrophils (FDR < 0.05). **e** Heatmap of DEGs associated with NF-κB signaling transduction. **f** GSEA demonstrating downregulation in inflammatory bowel disease (IBD)-associated and Toll-like receptor signaling pathways.** g** GSEA indicating suppression of endosomal trafficking, phagocytosis, and endocytosis pathways. **h** Heatmap of DEGs linked to endocytosis regulation. **i** Heatmap of significantly downregulated selected DEGs related to neutrophil reverse migration

We then investigated the molecular mechanism of itaconate in regulating neutrophil inflammation through an in vitro model. Several TLR agonists were tested for their ability to induce *Irg1* expression, and LPS was selected for subsequent experiments (Fig. S8a-b). Upon LPS stimulation, 4-OI significantly suppressed *Il-6* expression while up-regulating *Irg1* expression (Fig. S8c-d), indicating its functional efficacy. Initially, RNA-Seq analysis revealed that LPS stimulation significantly upregulated 1,172 genes and downregulated 1,182 genes compared to unstimulated controls (Fig. S9a). Both GO analysis and KEGG pathway enrichment for the significantly upregulated genes revealed that LPS stimulation resulted in neutrophil activated phenotype with robust inflammatory response (Fig. S9b-c). Subsequent analysis indicated that 4-OI treatment significantly upregulated 1,569 genes and downregulated 578 genes compared to LPS-stimulated neutrophils (Fig. S9d). Interestingly, heatmap analysis revealed that most of the genes downregulated by 4-OI treatment were those upregulated by LPS stimulation in neutrophils (Fig. 8a). Further GO analysis of the significantly downregulated genes after 4-OI treatment indicated that the related biological processes were mainly immune system process, innate immune response, inflammatory response, etc., while the related molecular functions were mainly pattern recognition receptor activity, coreceptor activity, etc. (Fig. 8b). Further KEGG analysis showed that the signaling pathways related to inflammation and immune response, such as JAK-STAT signaling pathway, cytokine-cytokine receptor interaction, Toll-like receptor signaling pathway, etc., were downregulated (Fig. 8c). Meanwhile, the significantly upregulated genes after 4-OI treatment were mainly enriched in items related to metabolism, transcriptional regulation, protein folding, etc. (Fig. S9e-f).

Further, Gene Set Enrichment Analysis (GSEA) was employed to explore the underlying mechanisms based on the 4-OI-responsive downregulated genes. The results revealed that the gene sets associated with inflammatory responses-including activation of innate immune response, canonical NF-κB signal transduction, positive regulation of IL-8 production, and cellular response to IFN-β-were enriched in LPS-stimulated neutrophils but depleted upon 4-OI treatment (Fig. 8d). Targeted analyses of the NF-κB signaling pathway and a select subset of NF-κB-related genes further revealed their down-regulation in 4-OI-treated neutrophils (Fig. 8e). Meanwhile, GSEA of relevant KEGG pathways also showed that gene sets linked to inflammatory bowel disease, Toll-like receptor signaling, leishmaniasis, and influenza A were less enriched in 4-OI-treated than in LPS-stimulated cells (Fig. 8f, Fig. S9g-h). Phagocytosis, endocytosis and other events are closely related to the functions of neutrophils. Through GSEA analysis, we found that the gene sets associated with endosome, phagocytic vesicle and endocytosis were enriched in LPS-stimulated cells, but absent within 4-OI treated cells (Fig. 8g), indicating that 4-OI can exert its anti-inflammatory effect by inhibiting the endocytosis- and phagocytosis-related processes in neutrophils. Subsequent targeted analyses of endocytosis and a select subset of endocytosis-related genes further revealed their downregulation upon 4-OI treatment (Fig. 8h). Consistent with these results, GSEA analysis also showed that 4-OI treatment inhibited Rab regulation of trafficking and neutrophil degranulation (Fig. S9i-j), which are all closely related to endocytosis of neutrophils. Furthermore, we performed a comparative analysis of the expression alterations of genes that are highly expressed in rM-ed neutrophils after 4-OI treatment. As depicted in Fig. 8i, numerous genes known to be highly expressed in rM-ed neutrophils were inhibited by 4-OI.

## Discussion

Recently, the *Irg1*/itaconate axis has gained significant attention as a key regulatory mechanism in inflammatory responses. Accumulating evidence suggests that this pathway represents a promising therapeutic target for IBD [22–24]. In this study, we employed DSS-induced mouse model of experimental colitis to demonstrate, for the first time, that the neutrophil-derived *Irg1*/itaconate axis exerts a critical protective effect during colitis progression. Our findings reveal that activation of this axis not only mitigates local intestinal tissue damage but also suppresses the accumulation of reverse migrated neutrophils in both peripheral blood and bone marrow, thereby facilitating the re-establishment of hematopoietic homeostasis.

For a significant period, research on the *Irg1*/itaconate axis has primarily focused on its regulatory effects in macrophages. These studies show that itaconate suppresses macrophage-mediated inflammation through multiple mechanisms, including activation of NRF2, inhibition of the NF-κB pathway, suppression of SDH and ROS production, and downregulation of aerobic glycolysis [31–34]. In recent years, the role of the *Irg1*/itaconate axis in neutrophils has received increasing attention. In the field of IBD research, earlier studies have shown that *Irg1* deficiency enhances the susceptibility of mice to experimental colitis [22]. More recently, exogenous itaconate treatment has been reported to significantly ameliorate colitis pathology, with proposed protective mechanisms by inhibiting pyroptosis in macrophages and intestinal epithelial cells [23]. In this study, using single-cell RNA sequencing analysis of mouse colitis tissues combined with immunofluorescence co-staining, we found that *Irg1* expression is predominantly upregulated in S100A8-positive neutrophils. Further investigation revealed that *Irg1* knockout mice exhibited increased vulnerability to colitis, accompanied by enhanced infiltration of activated neutrophils. Notably, neutrophil depletion experiments demonstrated exacerbated colitis severity in wild-type mice but alleviated pathology in *Irg1* knockout mice. Collectively, these findings indicate that neutrophils represent the primary cellular source of *Irg1*/itaconate in experimental colitis, and that the *Irg1*/itaconate axis plays an essential role in mediating neutrophil-dependent protective effects during colitis.

Patients with IBD often have extraintestinal manifestations, with anemia and peripheral osteoarthritis being common, but the pathological mechanism remains unclear [4, 5]. Studies have found that DSS-induced experimental colitis leads to myeloid-biased differentiation of bone marrow hematopoiesis, which in turn affects the level of MEP, thereby presenting systemic inflammation and anemia [35, 36]. Our research found that *Irg1*/itaconate axis deficiency shows higher levels of circulating neutrophils and more severe anemia, but bone marrow hematopoietic analysis shows that KO mice have lower GMP levels and higher MEP ratios. We speculate that this contradictory result between peripheral mature cells and bone marrow progenitor cells is due to the protective effect of a feedback mechanism triggered by the more severe inflammatory response caused by the absence of the *Irg1*/itaconate axis. How does colonic tissue damage affect hematopoiesis in bone marrow? We propose that the hematopoietic alterations induced by colitis arise from the synergistic effects of multiple factors. Impairment of intestinal barrier function permits translocation of noxious substances (e.g., endotoxins) into the systemic circulation. Concurrently, severe inflammation at the intestinal injury site drives elevated production of pro-inflammatory mediators including IL-1β, TNF-α, and G-CSF [6, 37–39]. Collectively, these stimuli act on hematopoietic stem cells, triggering emergency hematopoiesis and a bias toward myeloid differentiation. However, recent studies suggest that the reverse migration of neutrophils from the site of inflammatory injury may also be involved in the regulation of the hematopoietic system [13, 14, 40]. Interestingly, recent studies have found that neutrophils with high expression of *Irg1* in tendon injury models can home to the bone marrow and regulate hematopoiesis to control systemic inflammatory responses [29]. Some studies have shown that the *Irg1*/itaconate axis can promote the reverse migration of neutrophils to the peripheral blood [28]. We speculate that colonic injury may be involved in the regulation of bone marrow hematopoiesis through rM-ed neutrophils, and the results show that *Irg1* deficiency leads to a significant increase in the proportion of rM-ed neutrophils in the peripheral blood and bone marrow. Some studies have shown that in sepsis and aging conditions, reverse transendothelial migration of neutrophils can be recruited back to the lungs through the circulation, causing remote pulmonary inflammation and injury, thereby exacerbating systemic inflammation [27, 41, 42]. Combined with the antibody clearance experiment, it shows that *Irg1* deficiency neutrophils are effector cells that aggravate intestinal inflammatory responses and mucosal damage. We believe that the increase in reverse migrated *Irg1* deficiency neutrophils may spread inflammation to the periphery and bone marrow. These findings give rise to several questions that warrant further investigation. First, the mechanism underlying the increased abundance of rM-ed neutrophils in colitis models with *Irg1* deficiency remains poorly defined, and conflicting observations have been reported across distinct inflammatory model systems. While exogenous itaconate markedly suppresses the expression of genes associated with rM-ed neutrophils, these data require further validation and expansion. Second, the precise functional impact of rM-ed neutrophils on the hematopoietic system following their homing to the bone marrow necessitates rigorous experimental evaluation.

4-OI is a commonly used exogenous itaconic acid derivative with membrane-permeable properties. In our study, treatment of colitis in mice with 4-OI significantly alleviated intestinal damage and promoted the recovery of bone marrow hematopoietic homeostasis, suggesting that exogenous itaconate may serve as a promising therapeutic strategy for colitis. We observed that 4-OI treatment could significantly reduce the levels of rM-ed neutrophils in peripheral circulation and bone marrow. We speculate that this is closely related to its ability to promote the restoration of hematopoietic homeostasis in the bone marrow. However, the membrane-permeable nature of 4-OI means it can be taken up by various cells, and its good therapeutic effect should be a combined effect. This raises the possibility that 4-OI may directly regulate key cellular components such as intestinal epithelial cells and bone marrow hematopoietic stem and progenitor cells. To specifically assess the impact of 4-OI on activated neutrophils, we performed RNA sequencing on an in vitro model of LPS-stimulated neutrophils. Transcriptomic analysis revealed that 4-OI suppresses inflammatory signaling pathways including NF-κB and Toll-like receptor pathways, consistent with previous findings [21]. Notably, we also observed that 4-OI downregulates multiple genes associated with neutrophil reverse migration, a finding that aligns with the increased rM-ed neutrophil levels observed in *Irg1*-deficient models. More intriguingly, 4-OI treatment significantly suppressed the expression of genes involved in neutrophil functions such as endocytosis, phagocytosis, and degranulation, which are critical for pathogen clearance. Therefore, while their suppression may contribute to an anti-inflammatory phenotype, they may also increase the risk of infection dissemination. For instance, studies have shown that Staphylococcus aureus infection induces itaconate production but simultaneously impairs neutrophil bactericidal capacity by inhibiting glycolysis and oxidative burst activity [20]. Thus, when considering the therapeutic use of 4-OI in colitis, potential risks of infection spread must be carefully evaluated.

Taken together, this study has elucidated the pivotal protective role of the neutrophil *Irg1*/itaconate axis in experimental murine colitis. Our research has delineated an innovative functional paradigm of the *Irg1*/itaconate axis at the biological level. Specifically, at the site of intestinal injury, the presence of the *Irg1*/itaconate axis is crucial for determining the protective effects exerted by neutrophils. Regarding systemic alterations in the inflammatory response, the *Irg1*/itaconate axis may mitigate hematopoietic dysregulation by suppressing the levels of rM-ed neutrophils. At the translational medicine level, our findings demonstrate that exogenous 4-OI treatment can not only alleviate local intestinal inflammation but also facilitate the restoration of bone marrow hematopoietic homeostasis, indicating that itaconate and its derivatives are potential therapeutic targets for IBD with promising translational application prospects.

## Materials and methods

### Animals

*Irg1* knockout (KO) mice (S-KO-02680) were purchased from Cyagen Biotechnology. Male C57BL/6 J mice were obtained from SiPeiFu Biotechnology. All animals were housed under a 12 h light/dark cycle around 24℃in a SPF room with water and food provided ad libitum. All animal experiments and procedures in this study were approved by the Laboratory Animal Welfare and Ethics Committee of the Army Medical University (AMU).

### DSS-induced mouse colitis and 4-Octyl Itaconate treatment experimental procedures

To investigate the role of *Irg1*, colitis was induced in 6–8 weeks old wild-type (WT) and *Irg1* KO littermates with 3.0% DSS dissolved in drinking water given ad libitum for 7 consecutive days. To elucidate the efficacy of 4-OI in DSS-induced acute experimental colitis, male mice were administrated with 2.5% DSS via drinking water to induce light colitis and treated 4-OI (HY-112675, Med Chem Express) daily, commencing from day two and continuing until day 6 (25 mg/kg i.p. in 20% HP-β-CD) or vehicle (20% HP-β-CD in saline).

### Murine colonic single cells isolation and 10X Genomics sequencing

Colonic lamina propria mononuclear cells (LPMCs) from colitis mice were isolated as described previously [43]. In brief, the colon tissues were cut into 0.5 cm pieces in ice-cold HBSS (w/o). Tissue pieces were then pre-digested in EDTA-DTT buffer for 37 °C for 20 min, followed by further digestion with mixed solution (RPMI1640, 0.05% type II collagenase, 5% FBS, and DNAase 100 μg/mL) at 37 °C for 30 min. LPMCs were then isolated using a percoll gradient buffer and the single-cell suspension was obtained by filtering through a 40 μm cell filter. Cell viability was assessed by trypan blue staining, and samples with a viability rate > 90% were used for single-cell sequencing. Library construction and 10 × single-cell sequencing were performed according to the standard protocol provided by LC-Bio Technology (Hangzhou, China). Briefly, single cells were captured using the 10 × Genomics Chromium Single-Cell 3’ kit (V3) on a 10 × Chromium chip, and cDNA amplification and library construction were performed. The libraries were then sequenced on an Illumina NovaSeq6000 sequencing system by LC-Bio Technology.

### Disease activity index (DAI) score and histological analysis

The scoring criteria for the DAI have been previously described [44]. Briefly, DAI is mainly characterized by three parameters: weight loss index, rectal bleeding, and stool form, with each parameter scored from 0 to 4 (the maximum total score is 12). DAI scores were systematically quantified daily over a 7-day observation period to monitor colitis progression.

For histological analysis, the H&E images were scored by double-blind method as previously described [44]. The degree of damage to the distal colonic epithelium, including inflammatory cell infiltration (0–5), crypt damage (0–4), and ulceration (0–3), was evaluated respectively, and the sum of the scores was used as the histological score.

### Neutrophil depletion

The neutrophil depletion experiment was conducted with modifications based on previously reported methods [45]. One day prior to the administration of 3% DSS in drinking water, both *Irg1* KO and WT mice were intraperitoneally injected with either anti-Ly6G monoclonal antibody (mAb; catalog number 127650, clone: 1A8, Biolegend) or rat IgG2a isotype control mAb (catalog number 400566, clone: RTK2758, Biolegend) at a dosage of 200 μg per mouse every three days until the 6th day of the experiment. Then, they were switched to double distilled water for drinking until the 8th day for sample collection. The efficiency of neutrophil clearance was determined by flow cytometry in bone marrow and peripheral blood at days 0, 1, 3, 6, and 8, as well as in colon tissues at day 6 and the final time point.

### Immunofluorescence

Immunofluorescence (IF) staining was performed as previously described [46]. Detailed antibody information is provided in Table S1. The primary antibody concentrations were as follows: anti-IRG1 (1:200), anti-S100A8 (1:400), and anti-F4/80 (1:100). Fluorescence images were captured using a Thermo Fisher Evos M5000 microscope. Six 40 × high-power field images were taken from each sample, and semi-quantitative analysis of positive cells was performed using Image Pro Plus 6.0 software.

### Western blot

Western blots were carried out in accordance with our previous study [46]. Detailed information on the primary antibodies is shown in Supplemental Table 1. The Odyssey CLx (LI-COR Bioscience) was used to obtain infrared fluorescence images of the target protein bands, and band intensity was analyzed with Image Studio (Ver 5.2).

### Quantitative ream-time PCR

The relative mRNA expression of the genes mentioned in this study was detected using on a Bio-Rad CFX Connect Real-Time System with SYBR Premix (RR820A, TaKaRa) as we have previously reported [46]. The sequences of the primers used were listed in Table S2.

### ELISA assay

Colon tissue protein lysate supernatant was used for ELSA detection of cytokine levels according to the instructions. Detailed information of ELISA kits is shown in Table S3.

### Flow cytomsetry

For hematopoietic cell phenotype analysis, the HSPCs markers were Lineage^−^Sca1^+^c-Kit^+^ for LSK cells, Lineage^−^Sca1^−^c-Kit^+^ for myeloid progenitors (MPs), Lineage^−^Sca1^−^c-Kit^+^CD16/32^−^CD34^+^ for common myeloid progenitors (CMPs), Lineage^−^Sca1^−^c-Kit^+^CD16/32^−^CD34^−^ for megakaryocyte erythroid progenitors (MEPs), and Lineage^−^Sca1^−^c-Kit^+^CD16/32^+^CD34^+^ for granulocyte monocyte progenitors (GMPs) as previously described [47]. CXCR4^hi^ and CXCR2 ^hi^ Neutrophil subtypes in colon or bone marrow were analyzed as previously described [48]. The polymorphonuclear neutrophil (PMN) were defined as CD11b ^+^ Ly6G^+^ cells, the CD62L^+^ and the ICAM-1^hi^ CXCR1^lo^ rM-ed PMN within CD11b^+^Ly6G^+^ PMNs were detected as previously described [30]. The samples were detected with the Sony ID7000 and BD FAC Symphony flow cytometers. Antibody details are provided in Table S1 and the data were analyzed using Flow Jo 10.8.1.

### Isolation of bone marrow-derived neutrophil and treatment of LPS-stimulated neutrophil with 4-OI

Bone marrow-derived neutrophils were isolated with the Easy Sep Mouse neutrophil enrichment kit (19,762, STEM Cell) according to the manual protocols. After the viability examination, the neutrophils were suspended in IMDM medium and seeded into six-well plates at 4 × 10^6^ cells/well. The neutrophils were stimulated with LPS (93,572–42–0, Sigma) at a final concentration of 1 μg/ml for 4 h. 4-OI was dissolved in DMSO (67–68–5, 20 μM, Sigma) at a final concentration of 125 μM and added to the cells for an additional 2 h of treatment. Neutrophils were stimulated with distinct TLR agonists to investigate factors inducing *Irg1* upregulation; details are provided in Table S4. The cell sample were lysed with Trizol reagent, and then proceed with RNA-seq or qRT-PCR detection.

### RNA-seq analysis

Total RNA was extracted from neutrophil samples using Trizol reagent. The libraries were constructed using the VAHTS Universal V10 RNA-seq Library Prep Kit (Premixed Version) following the manufacturer's instructions. Transcriptome sequencing and analysis were conducted by OE Biotech Co., Ltd. (Shanghai, China). The libraries were sequenced on an Illumina Novaseq 6000 platform. Differential gene expression analysis was performed using DESeq2, with fold change > 2 or < 0.5 and *p* < 0.05 set as the threshold for significantly differentially expressed genes (DEGs). Subsequently, based on the hypergeometric distribution, GO and KEGG pathway enrichment analyses of DEGs were conducted to screen for significantly enriched terms. Meanwhile, gene set enrichment analysis (GSEA) was performed using GSEA_4.2.1 software and gene sets were obtained from the Molecular Signatures Database (MSigDB).

### Statistical analysis

Significant differences were calculated via GraphPad Prism 8.0. For the data of dynamic body weight measurement and DAI score, two-way repeated-measures ANOVA was conducted. For the data of colonic injury scores and neutrophil infiltration ratios of in bone marrow and colon, unpaired two-tailed Student's t tests were performed, with one-way ANOVA for multiple comparisons. A *P* value < 0.05 was considered statistically significant.

## Supplementary Information

Supplementary Material 1

Supplementary Material 2

## Data Availability

Single-cell RNA-seq data have been deposited in the NCBI GEO database under accession codes GSE312526 (https://www.ncbi.nlm.nih.gov/geo/query/acc.cgi?acc=GSE312526). RNA-seq data have been deposited in the NCBI GEO database under accession codes 312519 (https://www.ncbi.nlm.nih.gov/geo/query/acc.cgi?acc=GSE312519). All the other data generated, analyzed, or processed in this study are available upon reasonable request from the corresponding author.

## Abbreviations

Irg1: Immune response gene 1
IBD: Inflammatory bowel disease
4-OI: 4-Octyl itaconate
DSS: Dextran sulfate sodium
DAI: Disease activity index
GMP: Granulocyte-monocyte progenitors
MEP: Megakaryocyte-erythroid progenitors

## References

[1] Kaser A, Zeissig S, Blumberg RS. Inflammatory bowel disease. Annu Rev Immunol. 2010;28:573–621. 10.1146/annurev-immunol-030409-101225. 20192811 10.1146/annurev-immunol-030409-101225 PMC4620040

[2] de Souza HS, Fiocchi C. Immunopathogenesis of IBD: current state of the art. Nat Rev Gastroenterol Hepatol. 2016;13(1):13–27. 10.1038/nrgastro.2015.186. 26627550 10.1038/nrgastro.2015.186

[3] Maloy KJ, Powrie F. Intestinal homeostasis and its breakdown in inflammatory bowel disease. Nature. 2011;474(7351):298–306. 10.1038/nature10208. 21677746 10.1038/nature10208

[4] Rogler G, Singh A, Kavanaugh A, Rubin DT. Extraintestinal manifestations of inflammatory bowel disease: current concepts, treatment, and implications for disease management. Gastroenterology. 2021;161(4):1118–32. 10.1053/j.gastro.2021.07.042. 34358489 10.1053/j.gastro.2021.07.042 PMC8564770

[5] Gordon H, Burisch J, Ellul P, Karmiris K, Katsanos K, Allocca M, et al. ECCO guidelines on extraintestinal manifestations in inflammatory bowel disease. J Crohns Colitis. 2024;18(1):1–37. 10.1093/ecco-jcc/jjad108. 37351850 10.1093/ecco-jcc/jjad108

[6] Pietras EM, Mirantes-Barbeito C, Fong S, Loeffler D, Kovtonyuk LV, Zhang S, et al. Chronic interleukin-1 exposure drives haematopoietic stem cells towards precocious myeloid differentiation at the expense of self-renewal. Nat Cell Biol. 2016;18(6):607–18. 10.1038/ncb3346. 27111842 10.1038/ncb3346 PMC4884136

[7] Takizawa H, Manz MG. Impact of inflammation on early hematopoiesis and the microenvironment. Int J Hematol. 2017;106(1):27–33. 10.1007/s12185-017-2266-5. 28560577 10.1007/s12185-017-2266-5

[8] Hormaechea-Agulla D, Le DT, King KY. Common sources of inflammation and their impact on hematopoietic stem cell biology. Curr Stem Cell Rep. 2020;6(3):96–107. 10.1007/s40778-020-00177-z. 32837857 10.1007/s40778-020-00177-z PMC7429415

[9] Danne C, Skerniskyte J, Marteyn B, Sokol H. Neutrophils: from IBD to the gut microbiota. Nat Rev Gastroenterol Hepatol. 2024;21(3):184–97. 10.1038/s41575-023-00871-3. 38110547 10.1038/s41575-023-00871-3

[10] Chen T, Liu J, Hang R, Chen Q, Wang D. Neutrophils: from inflammatory bowel disease to colitis-associated colorectal cancer. J Inflamm Res. 2025;18:925–47. 10.2147/JIR.S497701. 10.2147/JIR.S497701 PMC11770381 39871958

[11] Zindl CL, Lai JF, Lee YK, Maynard CL, Harbour SN, Ouyang W, et al. IL-22-producing neutrophils contribute to antimicrobial defense and restitution of colonic epithelial integrity during colitis. Proc Natl Acad Sci USA. 2013;110(31):12768–73. 10.1073/pnas.1300318110. 23781104 10.1073/pnas.1300318110 PMC3732935

[12] Zhou G, Yu L, Fang L, Yang W, Yu T, Miao Y, et al. CD177(+) neutrophils as functionally activated neutrophils negatively regulate IBD. Gut. 2018;67(6):1052–63. 10.1136/gutjnl-2016-313535. 28468761 10.1136/gutjnl-2016-313535

[13] Xu Q, Zhao W, Yan M, Mei H. Neutrophil reverse migration. J Inflamm. 2022;19(1):22. 10.1186/s12950-022-00320-z. 10.1186/s12950-022-00320-z PMC9686117 36424665

[14] Ji J, Fan J. Neutrophil in reverse migration: role in sepsis. Front Immunol. 2021;12:656039. 10.3389/fimmu.2021.656039. 33790916 10.3389/fimmu.2021.656039 PMC8006006

[15] McGettrick AF, Bourner LA, Dorsey FC, O’Neill LAJ. Metabolic messengers: itaconate. Nat Metab. 2024;6(9):1661–7. 10.1038/s42255-024-01092-x. 39060560 10.1038/s42255-024-01092-x

[16] Peace CG, O'Neill LA. The role of itaconate in host defense and inflammation. J Clin Invest. 2022;132(2). 10.1172/JCI148548. 10.1172/JCI148548 PMC8759771 35040439

[17] Ye D, Wang P, Chen LL, Guan KL, Xiong Y. Itaconate in host inflammation and defense. Trends Endocrinol Metab. 2024;35(7):586–606. 10.1016/j.tem.2024.02.004. 38448252 10.1016/j.tem.2024.02.004

[18] Zhao Y, Liu Z, Liu G, Zhang Y, Liu S, Gan D, et al. Neutrophils resist ferroptosis and promote breast cancer metastasis through aconitate decarboxylase 1. Cell Metab. 2023;35(10):1688–703 e10. 10.1016/j.cmet.2023.09.004. 10.1016/j.cmet.2023.09.004 PMC10558089 37793345

[19] Wang C, Wen J, Yan Z, Zhou Y, Gong Z, Luo Y, et al. Suppressing neutrophil itaconate production attenuates Mycoplasma pneumoniae pneumonia. PLoS Pathog. 2024;20(11):e1012614. 10.1371/journal.ppat.1012614. 39499730 10.1371/journal.ppat.1012614 PMC11567624

[20] Tomlinson KL, Riquelme SA, Baskota SU, Drikic M, Monk IR, Stinear TP, et al. *Staphylococcus aureus* stimulates neutrophil itaconate production that suppresses the oxidative burst. Cell Rep. 2023;42(2):112064. 10.1016/j.celrep.2023.112064. 36724077 10.1016/j.celrep.2023.112064 PMC10387506

[21] Nair S, Huynh JP, Lampropoulou V, Loginicheva E, Esaulova E, Gounder AP, et al. Irg1 expression in myeloid cells prevents immunopathology during *M. tuberculosis* infection. J Exp Med. 2018;215(4):1035–45. 10.1084/jem.20180118. 29511063 10.1084/jem.20180118 PMC5881474

[22] Kim HW, Yu AR, Lee JW, Yoon HS, Lee BS, Park HW, et al. Aconitate decarboxylase 1 deficiency exacerbates mouse colitis induced by dextran sodium sulfate. Int J Mol Sci. 2022;23(8). 10.3390/ijms23084392. 10.3390/ijms23084392 PMC9025264 35457208

[23] Yang W, Wang Y, Wang T, Li C, Shi L, Zhang P, et al. Protective effects of IRG1/itaconate on acute colitis through the inhibition of gasdermins-mediated pyroptosis and inflammation response. Genes Dis. 2023;10(4):1552–63. 10.1016/j.gendis.2022.05.039. 37397544 10.1016/j.gendis.2022.05.039 PMC10311025

[24] Wang Y, Zhao X, Gao Y, Zhao C, Li J, Wang S, et al. 4-octyl itaconate alleviates dextran sulfate sodium-induced ulcerative colitis in mice via activating the KEAP1-NRF2 pathway. Inflammopharmacology. 2024;32(4):2555–74. 10.1007/s10787-024-01490-3. 38767761 10.1007/s10787-024-01490-3

[25] Frede A, Czarnewski P, Monasterio G, Tripathi KP, Bejarano DA, Ramirez Flores RO, et al. B cell expansion hinders the stroma-epithelium regenerative cross talk during mucosal healing. Immunity. 2022;55(12):2336–51 e12. 10.1016/j.immuni.2022.11.002. 10.1016/j.immuni.2022.11.002 36462502

[26] Colom B, Bodkin JV, Beyrau M, Woodfin A, Ody C, Rourke C, et al. Leukotriene B4-neutrophil elastase axis drives neutrophil reverse transendothelial cell migration in vivo. Immunity. 2015;42(6):1075–86. 10.1016/j.immuni.2015.05.010. 26047922 10.1016/j.immuni.2015.05.010 PMC4504024

[27] Jin H, Aziz M, Ode Y, Wang P. CIRP induces neutrophil reverse transendothelial migration in sepsis. Shock. 2019;51(5):548–56. 10.1097/SHK.0000000000001257. 30148763 10.1097/SHK.0000000000001257 PMC6387861

[28] Ji J, Zhong H, Li Y, Billiar TR, Wilson MA, Scott MJ, et al. *IRG1/ACOD1* promotes neutrophil reverse migration and alleviates local inflammation. J Leukoc Biol. 2024;116(4):854–63. 10.1093/jleuko/qiae110. 38713770 10.1093/jleuko/qiae110 PMC11444257

[29] Crossley JL, Ostashevskaya-Gohstand S, Comazzetto S, Hook JS, Guo L, Vishlaghi N, et al. Itaconate-producing neutrophils regulate local and systemic inflammation following trauma. JCI insight. 2023;8(20). 10.1172/jci.insight.169208. 10.1172/jci.insight.169208 PMC10619500 37707952

[30] Zi SF, Wu XJ, Tang Y, Liang YP, Liu X, Wang L, et al. Endothelial cell-derived extracellular vesicles promote aberrant neutrophil trafficking and subsequent remote lung injury. Adv Sci. 2024. 10.1002/advs.202400647. 10.1002/advs.202400647 PMC11481253 39119837

[31] Mills EL, Ryan DG, Prag HA, Dikovskaya D, Menon D, Zaslona Z, et al. Itaconate is an anti-inflammatory metabolite that activates Nrf2 via alkylation of KEAP1. Nature. 2018;556(7699):113–7. 10.1038/nature25986. 29590092 10.1038/nature25986 PMC6047741

[32] Bambouskova M, Gorvel L, Lampropoulou V, Sergushichev A, Loginicheva E, Johnson K, et al. Electrophilic properties of itaconate and derivatives regulate the IκBζ-ATF3 inflammatory axis. Nature. 2018;556(7702):501–4. 10.1038/s41586-018-0052-z. 29670287 10.1038/s41586-018-0052-z PMC6037913

[33] Chen F, Elgaher WAM, Winterhoff M, Bussow K, Waqas FH, Graner E, et al. Citraconate inhibits ACOD1 (IRG1) catalysis, reduces interferon responses and oxidative stress, and modulates inflammation and cell metabolism. Nat Metab. 2022;4(5):534–46. 10.1038/s42255-022-00577-x. 35655026 10.1038/s42255-022-00577-x PMC9170585

[34] Liao ST, Han C, Xu DQ, Fu XW, Wang JS, Kong LY. 4-octyl itaconate inhibits aerobic glycolysis by targeting GAPDH to exert anti-inflammatory effects. Nat Commun. 2019;10(1):5091. 10.1038/s41467-019-13078-5. 31704924 10.1038/s41467-019-13078-5 PMC6841710

[35] Fu N, Wu F, Jiang Z, Kim W, Ruan T, Malagola E, et al. Acute intestinal inflammation depletes/recruits histamine-expressing myeloid cells from the bone marrow leading to exhaustion of MB-HSCs. Cell Mol Gastroenterol Hepatol. 2021;11(4):1119–38. 10.1016/j.jcmgh.2020.11.007. 33249238 10.1016/j.jcmgh.2020.11.007 PMC7903065

[36] Sezaki M, Hayashi Y, Nakato G, Wang Y, Nakata S, Biswas S, et al. Hematopoietic stem and progenitor cells integrate microbial signals to promote post-inflammation gut tissue repair. EMBO J. 2022;41(22):e110712. 10.15252/embj.2022110712. 36254590 10.15252/embj.2022110712 PMC9670188

[37] Noel JG, Ramser SW, Pitstick L, Goetzman HS, Dale EL, Potter A, et al. IL-1/MyD88-dependent G-CSF and IL-6 secretion mediates postburn anemia. J Immunol. 2023;210(7):972–80. 10.4049/jimmunol.2200785. 36779805 10.4049/jimmunol.2200785 PMC10038902

[38] Kovtonyuk LV, Caiado F, Garcia-Martin S, Manz EM, Helbling P, Takizawa H, et al. IL-1 mediates microbiome-induced inflammaging of hematopoietic stem cells in mice. Blood. 2022;139(1):44–58. 10.1182/blood.2021011570. 34525198 10.1182/blood.2021011570

[39] Wang JX, Saijo K, Skola D, Jin C, Ma Q, Merkurjev D, et al. Histone demethylase LSD1 regulates hematopoietic stem cells homeostasis and protects from death by endotoxic shock. Proc Natl Acad Sci USA. 2018;115(2):E244–52. 10.1073/pnas.1718759114. 29263096 10.1073/pnas.1718759114 PMC5777077

[40] Wang J, Hossain M, Thanabalasuriar A, Gunzer M, Meininger C, Kubes P. Visualizing the function and fate of neutrophils in sterile injury and repair. Science. 2017;358(6359):111–5. 10.1126/science.aam9690. 28983053 10.1126/science.aam9690

[41] Ji J, Zhong H, Wang Y, Liu J, Tang J, Liu Z. Chemerin attracts neutrophil reverse migration by interacting with C-C motif chemokine receptor-like 2. Cell Death Dis. 2024;15(6):425. 10.1038/s41419-024-06820-5. 38890311 10.1038/s41419-024-06820-5 PMC11189533

[42] Barkaway A, Rolas L, Joulia R, Bodkin J, Lenn T, Owen-Woods C, et al. Age-related changes in the local milieu of inflamed tissues cause aberrant neutrophil trafficking and subsequent remote organ damage. Immunity. 2021;54(7):1494–510 e7. 10.1016/j.immuni.2021.04.025. 10.1016/j.immuni.2021.04.025 PMC8284598 34033752

[43] Lu HY, Zhang C, Wu W, Chen HM, Lin RT, Sun RC, et al. MCPIP1 restrains mucosal inflammation by orchestrating the intestinal monocyte to macrophage maturation via an ATF3-AP1S2 axis. Gut. 2023;72(5):882–95. 10.1136/gutjnl-2022-327183. 37015751 10.1136/gutjnl-2022-327183

[44] Zhao N, Wang G, Long S, Liu D, Gao J, Xu Y, et al. Neutrophils-derived Spink7 as one safeguard against experimental murine colitis. Biochimica et Biophysica Acta (BBA). 2021;1867(6):166125. 10.1016/j.bbadis.2021.166125. 10.1016/j.bbadis.2021.166125 33722746

[45] Wang X, Cai J, Lin B, Ma M, Tao Y, Zhou Y, et al. GPR34-mediated sensing of lysophosphatidylserine released by apoptotic neutrophils activates type 3 innate lymphoid cells to mediate tissue repair. Immunity. 2021;54(6):1123–36 e8. 10.1016/j.immuni.2021.05.007. 10.1016/j.immuni.2021.05.007 34107271

[46] Zhao N, Wang G, Long S, Lv X, Ran X, Wang J, et al. The antiprotease Spink7 promotes inflammation resolution by modulating multiple proteases activities during wound healing. Clin Transl Med. 2025;15(4):e70291. 10.1002/ctm2.70291. 40147022 10.1002/ctm2.70291 PMC11949503

[47] Hu MJ, Chen NC, Chen M, Chen F, Lu YK, Xu Y, et al. Transcription factor Nkx2-3 maintains the self-renewal of hematopoietic stem cells by regulating mitophagy. Leukemia. 2023;37(6):1361–74. 10.1038/s41375-023-01907-y. 37095209 10.1038/s41375-023-01907-y

[48] Xie X, Shi Q, Wu P, Zhang X, Kambara H, Su J, et al. Single-cell transcriptome profiling reveals neutrophil heterogeneity in homeostasis and infection. Nat Immunol. 2020;21(9):1119–33. 10.1038/s41590-020-0736-z. 32719519 10.1038/s41590-020-0736-z PMC7442692

