# Supplementary material for: Neutrophil Irg1/itaconate axis protects against experimental colitis by suppressing local inflammation and maintaining hematopoietic homeostasis
Source: Mol Biomed. 2025 Dec 19;6:143. doi: 10.1186/s43556-025-00390-4 (PMC12717350; doi:10.1186/s43556-025-00390-4)
Supplement: Supplementary file 1 — Supplementary Material 1 [file 43556_2025_390_MOESM1_ESM.docx]

Journal: Molecular Biomedicine

Neutrophil *Irg1*/itaconate axis protects against experimental colitis by suppressing local inflammation and maintaining hematopoietic homeostasis

Na Zhao^1,^^2^, Guojian Wang^1^, Shuang Long^1^, Yin Chen1, Jining Gao^1^, Xiaofan Lv^1^, Xinze Ran^1^, Yi Jia^2^*****, Tao Wang^1^*****.

^1^ Institute of Combined Injury, Chongqing Engineering Research Center for Nanomedicine, School of Preventive Military Medicine, Army Medical University (Third Military Medical University), Chongqing 400038, China. ^2^ Institute of Materia Medica and Department of Pharmaceutics, College of Pharmacy, Army Medical University (Third Military Medical University), Chongqing, 400038, China.

***Correspondence:** Tao Wang, e-mail: [wangtmmu@hotmail.com](mailto:wangtmmu@hotmail.com), ORCID: <https://orcid.org/0000-0003-0975-8072>. Yi Jia, e-mail: [jy@tmmu.edu.cn](mailto:jy@tmmu.edu.cn).

**Supplementary figure**


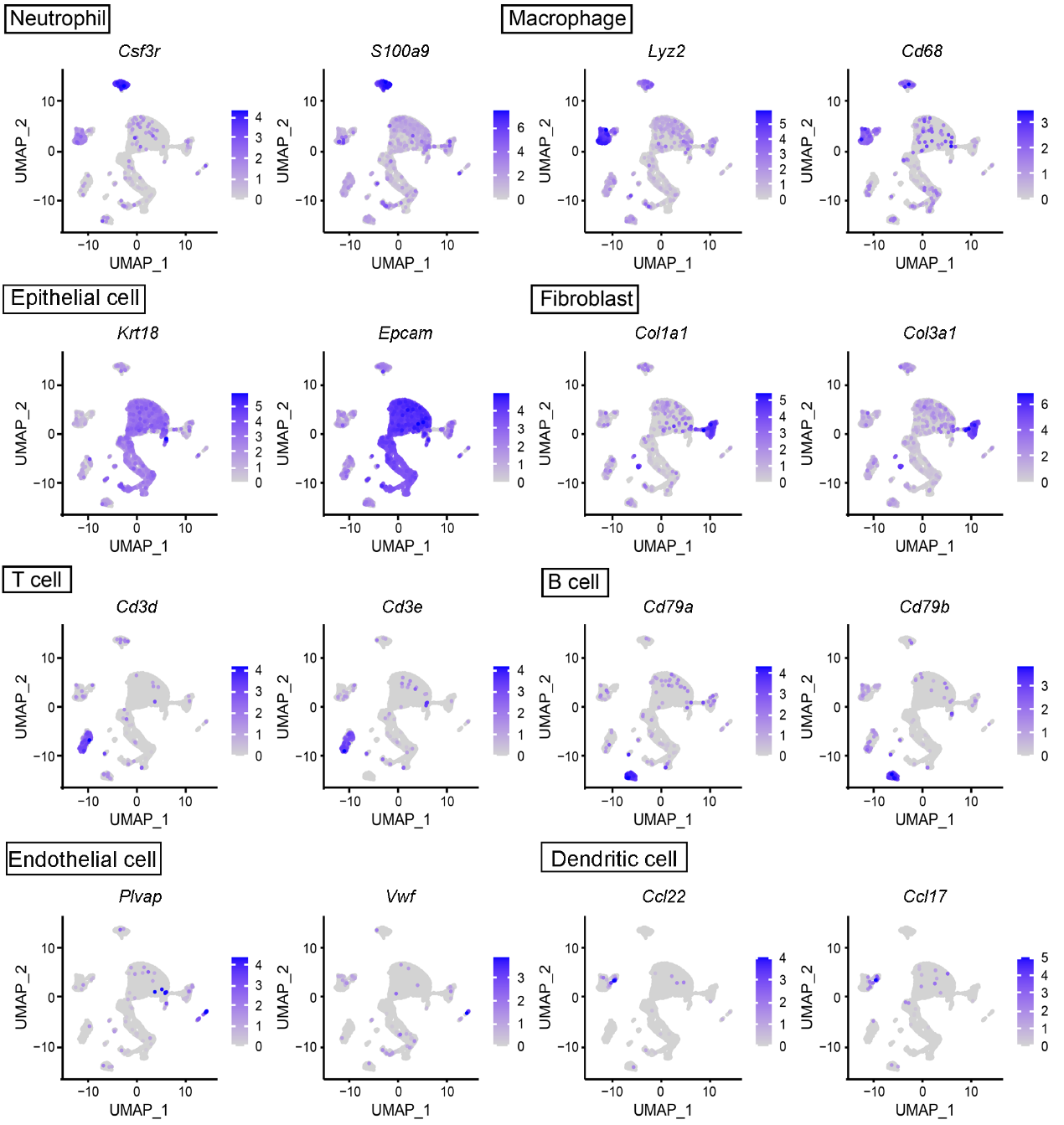


**Figure S1. Single R-based cell type annotation using marker genes in DSS-induced colitis.** Single-cell RNA sequencing analysis of colonic tissues from mice with 7-day DSS-induced colitis. Cell clusters were annotated using Single R package with canonical marker genes. Representative UMAP plot shows major immune and stromal populations.


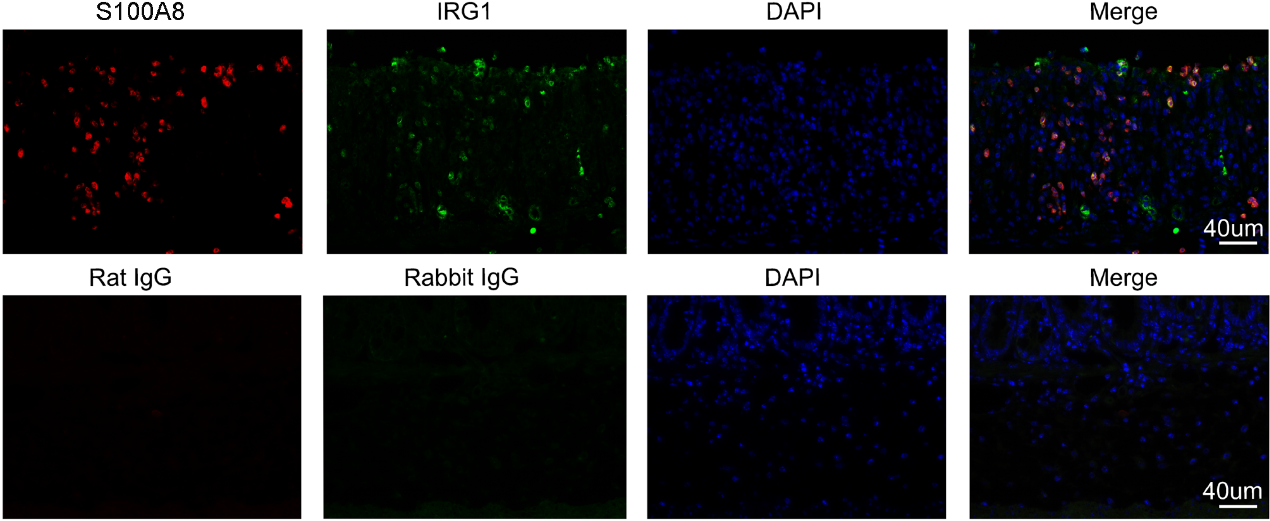


**Figure S2. IRG1 expression in colonic neutrophils.** Representative immunofluorescence images showing IRG1 (green) and S100A8 (red) co-staining with isotype control in colons of 7-day DSS-induced colitis mice.


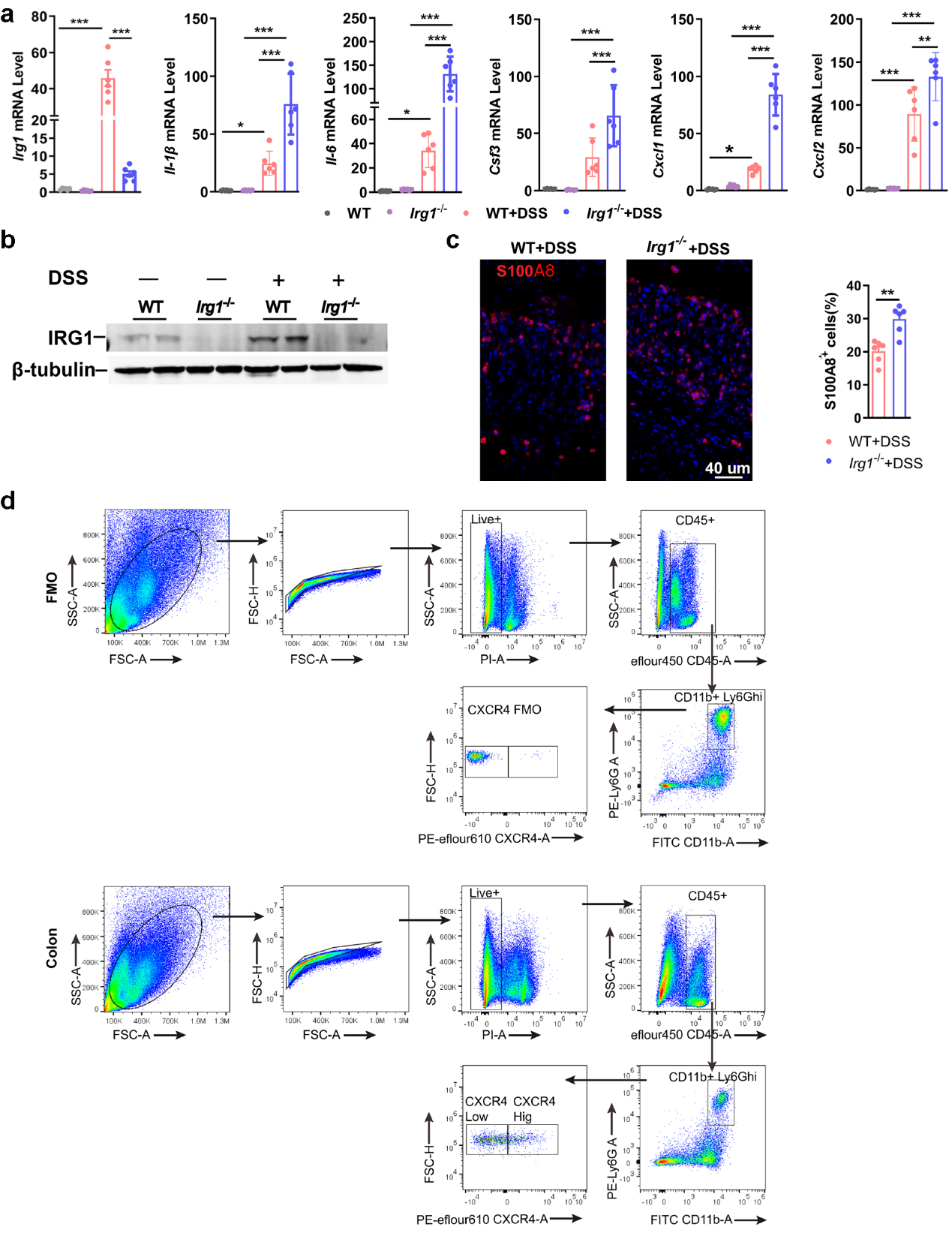


**Figure S3. *Irg1* deficiency exacerbates inflammation in DSS-induced colitis. a.** mRNA expression of *Irg1* and inflammatory factors (*Il-1β, Il-6, Csf3, Cxcl1and Cxcl2*) in colon tissues of wild-type (WT) and *Irg1^-/-^* mice at day 7 post-DSS (n = 6 samples/group). **b.** IRG1 protein expression (western blot) at day 7 post-DSS. **c**. IRG1 protein expression (western blot) in WT versus *Irg1* KO colon tissues at day 7 post-DSS. **d**. Immunofluorescent staining for neutrophils (S100A8, Left) or (semi-quantitative analysis, Right) in colon at day 7 post-DSS. **e.** Gating strategy for CXCR4^hi^ and CXCR4^lo^ neutrophils in colonic suspensions at day 7 post-DSS. Data are represented as the mean ± SD. *^∗^p* < 0.05, *^∗∗^p* < 0.01, *^∗∗∗^p* < 0.001.


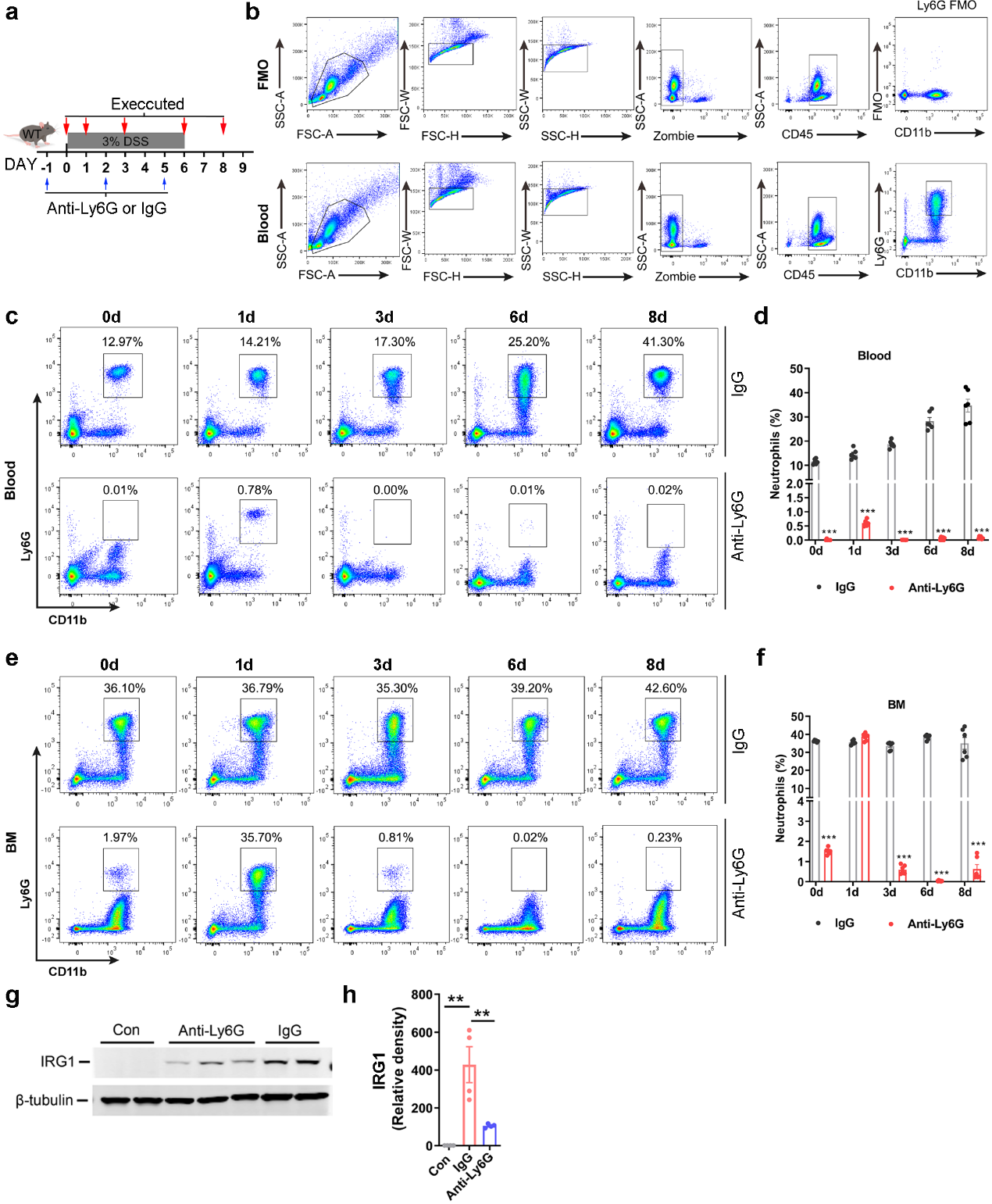


**Figure S4. Neutrophil depletion reduces IRG1 expression in DSS colitis.**

**a.** Experimental timeline schematic for neutrophil depletion and flow cytometry analysis. **b.** Gating strategy for neutrophils in blood of WT mice at day 6 post-DSS induction. **c.** Representative flow cytometry plots showing Ly6G-mediated neutrophil depletion in blood of WT mice with DSS-colitis at indicated timepoints. **d.** Neutrophil proportions in blood after anti-Ly6G depletion at serial timepoints (n = 6 samples/group). **e.** Representative flow cytometry plots of neutrophil depletion in bone marrow of WT DSS-colitis mice at indicated timepoints. **f.**  Neutrophil proportions in bone marrow after anti-Ly6G depletion (n = 6 samples/group). **g.** Representative western blot of IRG1 protein expression in colonic tissues of WT mice at day 8 post-DSS. **h.** Quantification of IRG1 protein levels in colon tissues at day 8 post-DSS (n = 4 samples/group). Data are represented as the mean ± SD. *^∗^ p* < 0.05, *^∗∗^ p* < 0.01, *^∗∗∗^ p* < 0.001 by Student's t-test.


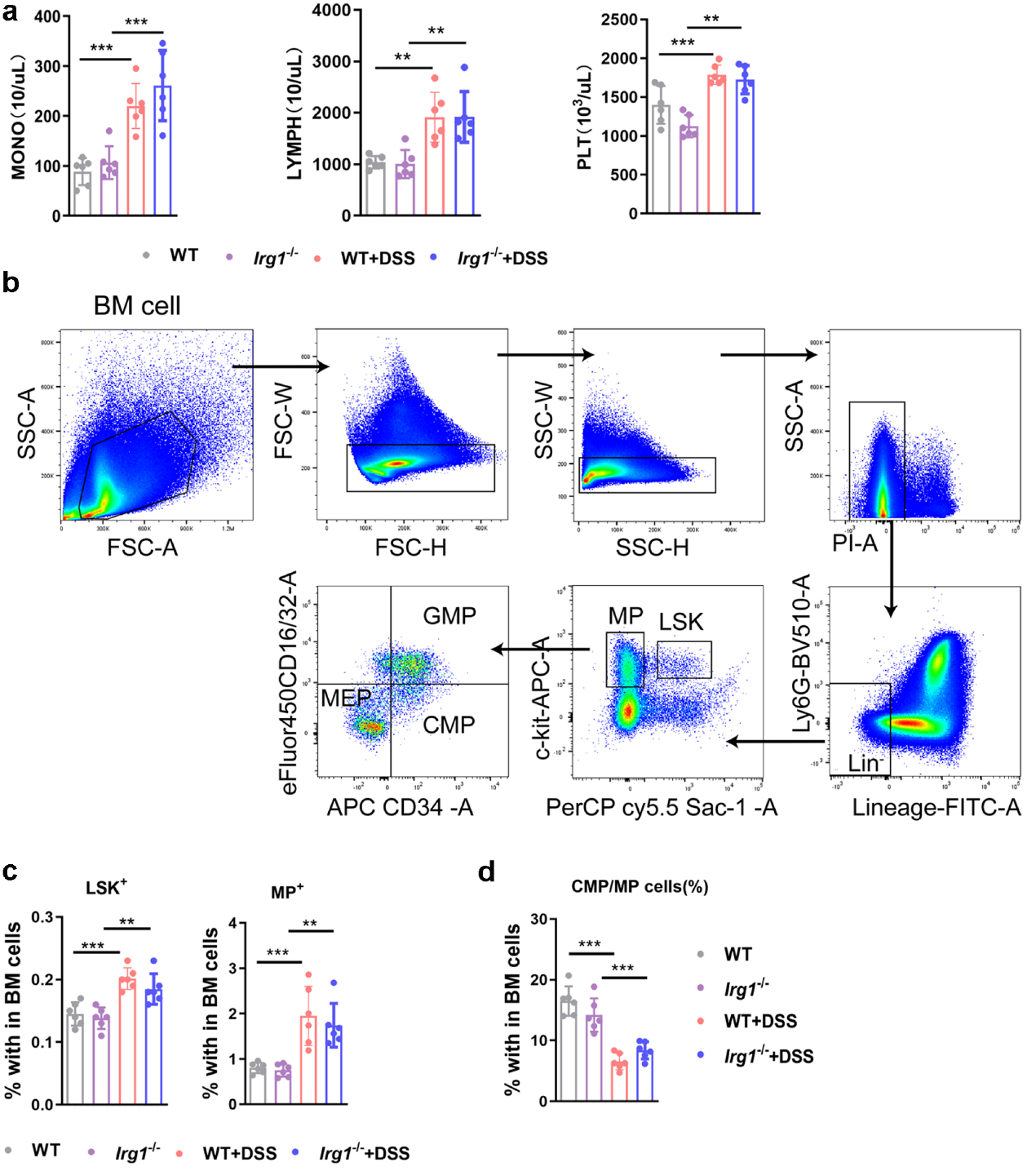


**Figure S5.** **Hematopoietic alterations in DSS-induced colitis.**

**a**. Peripheral blood cell counts at day 7 of DSS colitis in WT and *Irg1*^-/-^ mice. **b**. Gating strategy for bone marrow HSPCs. **c**. Frequency of LSK (left) and MP (right) populations in bone marrow (single-cell suspensions from one femur and tibia) at day 7 post-DSS (n = 6 samples/group). d**.** Frequency of CMP populations in bone marrow. Data are represented as the mean ± SD. *^∗^ p* < 0.05, *^∗∗^ p* < 0.01, *^∗∗∗^ p* < 0.001.


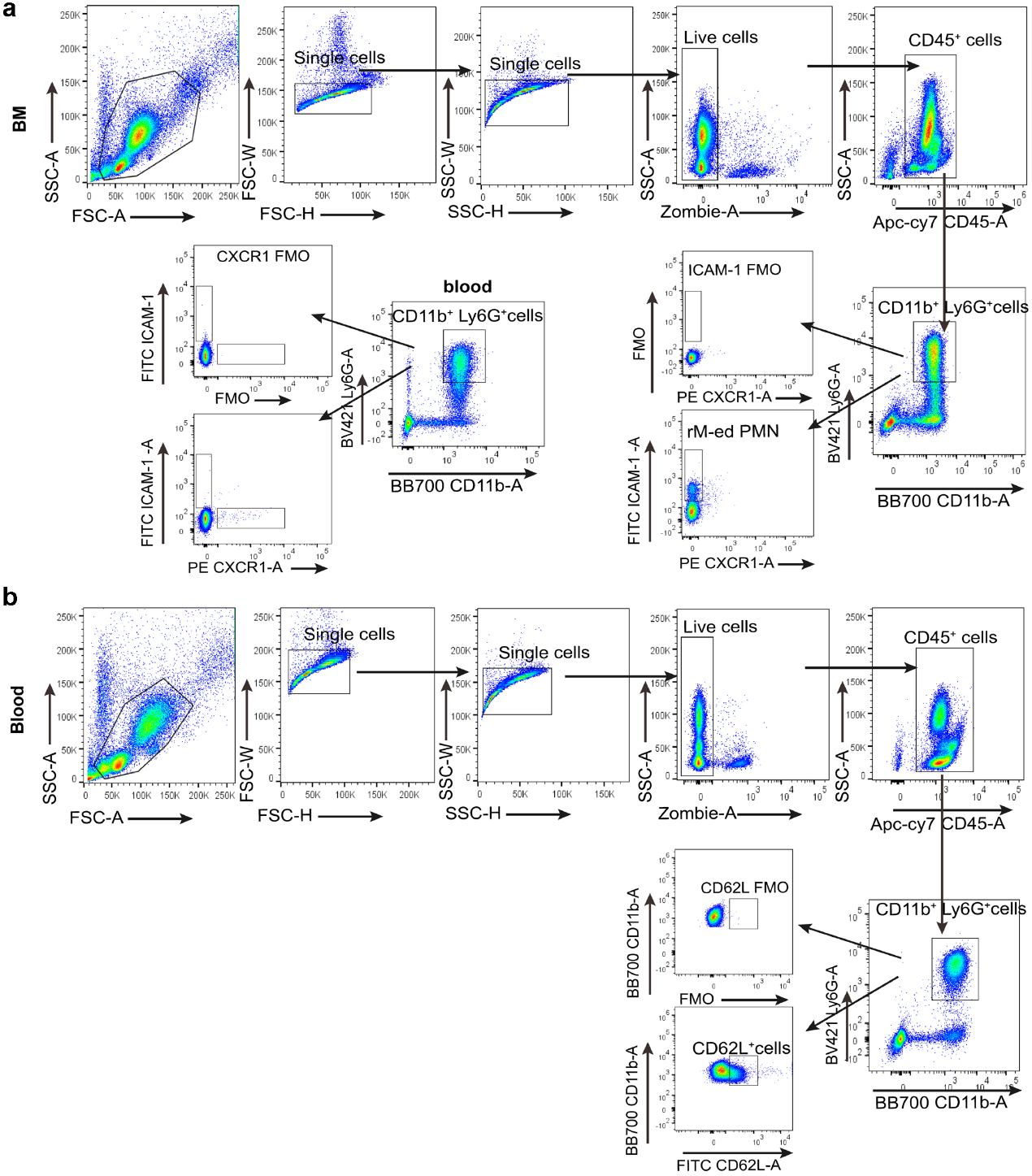


**Figure S6.** **Gating strategy for rM-ed and CD62L PMN in mice.**

**a.** Gating strategy for rM-ed PMNs in bone marrow at day 7 of DSS induction. **b.** Gating strategy analyzing CD62L^+^/CD11b^+^ PMNs in blood at day 7 post-DSS induction.


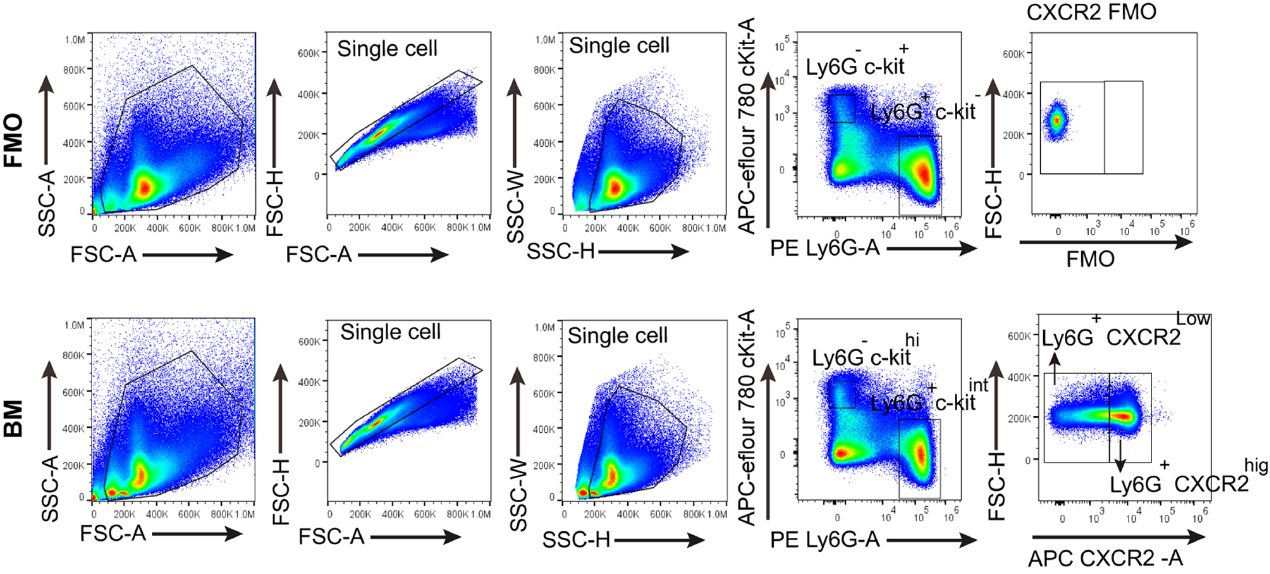


**Figure S7. Gating strategy for identification of CXCR2^hi^ and CXCR2^lo^**

**neutrophil subsets in bone marrow.**
Representative flow cytometry plots showing CXCR2 expression profiles in bone marrow from WT mice at day 7 post-DSS-induced colitis.


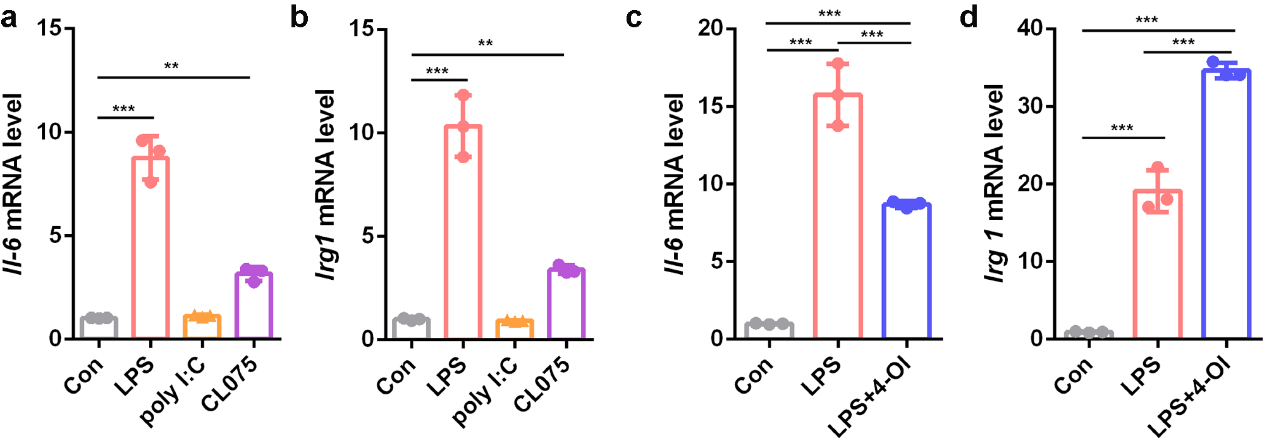


**Figure S8. Upregulation of Irg1 mRNA expression by TLR agonists and 4-OI.**

**a and b.** mRNA expression levels of *Il-6* and *Irg1* in bone marrow-derived neutrophils (BMNs) after 6 h stimulation with TLR agonists: LPS (500ng/mL), poly I:c(5μg/mL) and CL075 (5μM) (n = 3).

**c and d.** *Il-6* (c) and *Irg1* (d) mRNA expression in BMNs untreated or 4-OI-treated(125μM,2h) after stimulated by LPS(1μg/mL) for 4 h (n = 3).Data are represented as the mean ± SD. *^∗^ p* < 0.05, *^∗∗^ p* < 0.01, *^∗∗∗^ p* < 0.001.


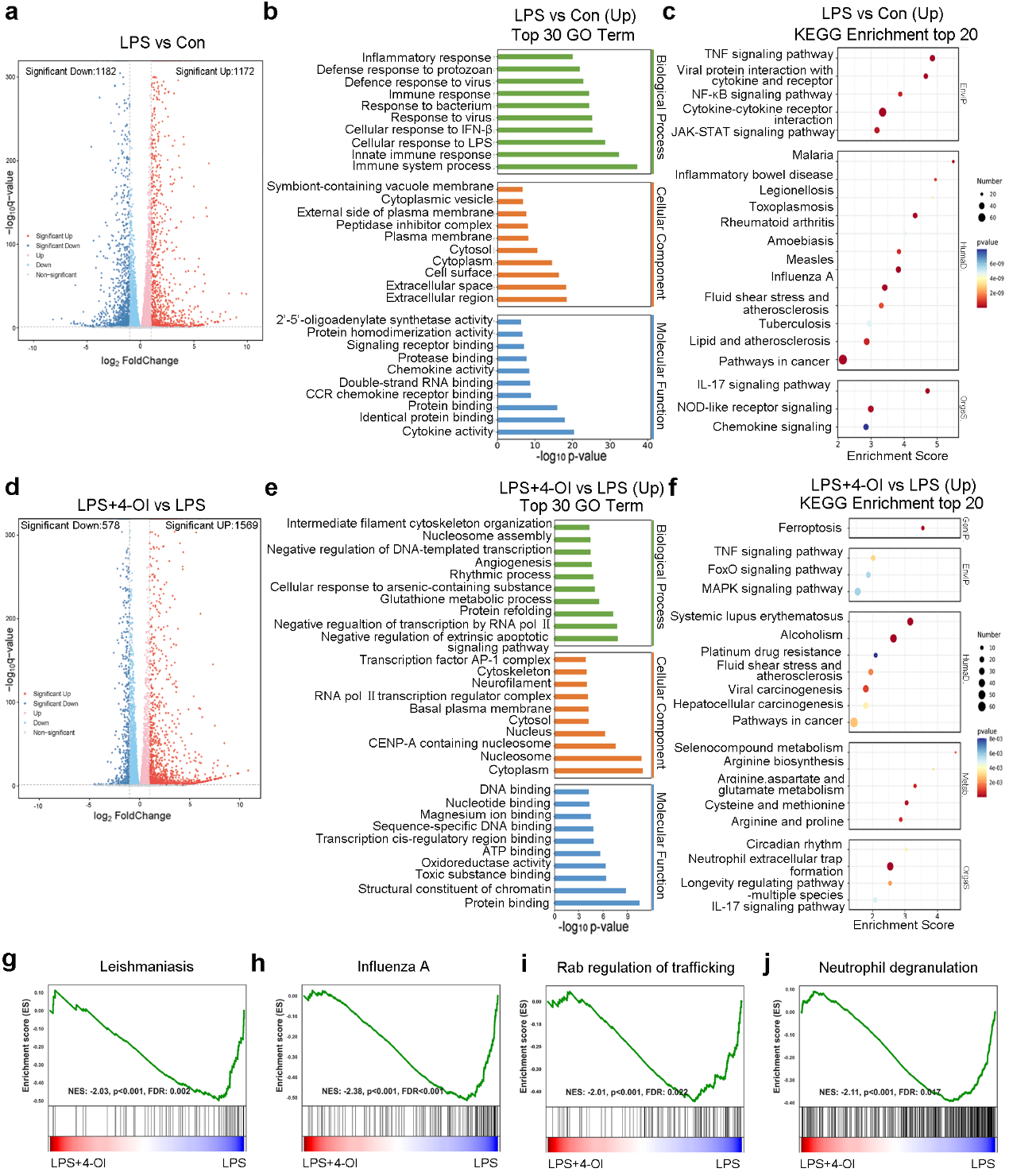


**Figure S9. Transcriptomic profiling of 4-OI-treated neutrophils.** **a**. Volcano plot of DEGs in LPS-stimulated vs. untreated neutrophils (fold change≥ 2.0, *P* < 0.05). **b**. Top 30 significantly enriched GO terms for upregulated DEGs in LPS vs. untreated neutrophils. **c**. Top 20 enriched KEGG pathways in upregulated DEGs. **d**. Volcano plot of DEGs in 4-OI + LPS vs. LPS alone neutrophils. (fold change≥ 2.0, *P* < 0.05). **e**. Top 30 significantly enriched GO terms for upregulated DEGs in 4-OI + LPS vs. LPS alone neutrophils. **f**. Top 20 KEGG pathways enriched in upregulated DEGs in 4-OI + LPS vs. LPS alone. **g** and **h**. GSEA showing significant suppression of Rab regulation of vesicle trafficking (**g**) and neutrophil degranulation pathways (**h**) in 4-OI-treated neutrophils vs. LPS-stimulated cells. **i** and **j** Significant attenuation of Influenza A virus infection (**i**) and Leishmania infection response pathways (**j**) in 4-OI-treated neutrophils versus LPS-stimulated neutrophils.

**Supplementary Table S1**

**Antibodies used for FC, WB or IF**

| **Antibody** | **Source** | **Cat. No** | **Assay** |
| --- | --- | --- | --- |
| FITC anti-mouse Lineage Cocktail | Thermo Fisher | 22-7770-72 | FC |
| APC-eFluor780 anti-mouse c-Kit | Thermo Fisher | 47-1171-82 | FC |
| PerCP-Cyanine5.5 anti-mouse Sca-1 | Thermo Fisher | 45-5981-82 | FC |
| eFluor 660 anti-mouse CD34 | Thermo Fisher | 50-0341-82 | FC |
| eFluor 450 anti-mouse CD16/32 | Thermo Fisher | 48-0161-82 | FC |
| PI | Biolegend | 640914 | FC |
| APC-Cy7 anti-mouse CD45 | BioLegend | 103116 | FC |
| eFluor 450 anti-mouse CD45 | Thermo Fisher | 48-0459-42 | FC |
| FITC anti-Mouse CD11b | Biolegend | 101205 | FC |
| PE anti-mouse Ly6G | Biolegend | 127608 | FC |
| PerCP-Cyanine5.5 anti-mouse CD11b | Thermo Fisher | 45-0112-82 | FC |
| APC anti-mouse CXCR2 | BioLegend | 149312 | FC |
| PE-eFluor 610 CXCR4 | Thermo Fisher | 61-9991-82 | FC |
| eFluor 506 anti-mouse Ly6G | Thermo Fisher | 69-5931-82 | FC |
| BV421 anti-mouse Ly6G | Invitrogen | 404-5931-80 | FC |
| PE anti-mouse CXCR1 | R&D | FAB8628P-025 | FC |
| FITC anti-mouse ICAM-1/CD54 | Elabscience | E-ABF1018C | FC |
| FITC anti-mouse ICAM-1/CD54 | Biolegend | 116105 | FC |
| PE anti-mouse CD181(CXCR1) | BD | 566383 | FC |
| [FITC Anti-Mouse CD62L](https://gys.tmmu.edu.cn/order/snapshot?id=1519935) | BD | 561917 | FC |
| BV421 anti-mouse Ly6G | BD | 562737 | FC |
| BB700 Anti-Mouse CD11b | BD | 566416 | FC |
| Zombine | Biolegend | 423110 | FC |
| PE Rat IgG2a Isotype Control[2A3] | Elabscience | E-AB-F09833D | FC |
| PE-EF610 Rat IgG 2b | 423110 | 61-4031-80 | FC |
| APC Rat IgG2a | Biolegend | 400511 | FC |
| FITC Rat IgG2b | Thermo Fisher | PA5-33225 | FC |
| InVivoMAb anti-mouse Ly-6G | Biolegend | 127649 | InVivo |
| Rat IgG2a | Biolegend | 400565 | InVivo |
| Anti-IRG1 antibody | Abcam | ab222411 | WB |
| Anti-IRG1 antibody | Cell Signaling Technology | 17805S | IF |
| Anti-S100A8 | Invitrogen | MA5-24066 | IF |
| Anti-β-tubulin | Beyotime | AF1216 | WB |
| Anti-F4/80 | Biolegend | 123101 | IF |
| Normal rat IgG | Thermo Fisher | 14-4031-82 | IF |
| Normal Rabbit IgG | Sigma | 12-370 | IF |
| Anti-rat IgG (H+L), (Alexa Fluor ® 555 Conjugate) | Cell Signaling Technology | 4417S | IF |
| Anti-rabbit IgG (H+L), (Alexa Fluor® 488 Conjugate) | Cell Signaling Technology | 4412S | IF |

FC, flow cytometry; WB, Western blot; IF, Immunofluorescence.

**Supplementary Table S2**

**Primers for mRNA expression analysis in mice**

| **Gene** | **Forward primer (5’-3’)** | **Reverse primer (5’-3’)** |
| --- | --- | --- |
| *Irg1* | GGT ATC ATT CGG AGG AGC AAG AG | ACA GTG CTG GAG GTG TTG GAA C |
| *Il-1β* | TCTCGCAGCAGCACATCA | CACACACCAGCAGGTTAT |
| *IL6* | TGGGAAATCGTGGAAATGAG | CTCTGAAGGACTCTGGCTTTG |
| *TNFα* | CCCGGGCTCAGCCTCTTCTCATTC | GGATCCGGTGGTTTGCTACGACGT |
| *Cxcl1* | CTGGGATTCACCTCAAGAACATC | CAGGGTCAAGGCAAGCCTC |
| *Cxcl2* | CCTGCCAAGGGTTGACTTCA | TTTTGACCGCCCTTGAGAGT |
| *G-csf* | ATGGCTCAACTTTCTGCCCAG | CTGACAGTGACCAGGGGAAC |
| *TBP* | AAGGGAGAATCATGGACCAG | CCGTAAGGCATCATTGGACT |
| *β-actin* | GGCTGTATTCCCCTCCATCG | CCAGTTGGTAACAATGCCATGT |

**Supplementary Table S3**

**Cytokines for ELISA analysis**

| **Cytokines** | **Source** | **Cat. No** |
| --- | --- | --- |
| Mouse IL-1β | Multi sciences | EK201B/3-96 |
| Mouse IL-6 | 4A Biotech | CME0006 |
| Mouse TNF-α | 4A Biotech | CME0004 |
| Mouse G-CSF | Multi sciences | EK269 |
| Mouse CXCL1/KC | Multi sciences | EK296 |

**Supplementary Table S4**

**TLR agonists that induced *Irg1* expression**

| **Compound** | **Source** | **Target** | **Cat. No** |
| --- | --- | --- | --- |
| CL075 | MCE | TLR8 | HY-117066 |
| Poly (I:C): Kanamycin | MCE | TLR3 | HY-107202A |
| LPS | Sigma | TLR4 | 93572-42-0 |
