# Supplementary material for: Neutrophil Irg1/itaconate axis protects against experimental colitis by suppressing local inflammation and maintaining hematopoietic homeostasis
Source: Mol Biomed. 2025 Dec 19;6:143. doi: 10.1186/s43556-025-00390-4 (PMC12717350; doi:10.1186/s43556-025-00390-4)
Supplement: Supplementary file 2 — Supplementary Material 2 [file 43556_2025_390_MOESM2_ESM.docx]

Uncropped Western blotting membrane images


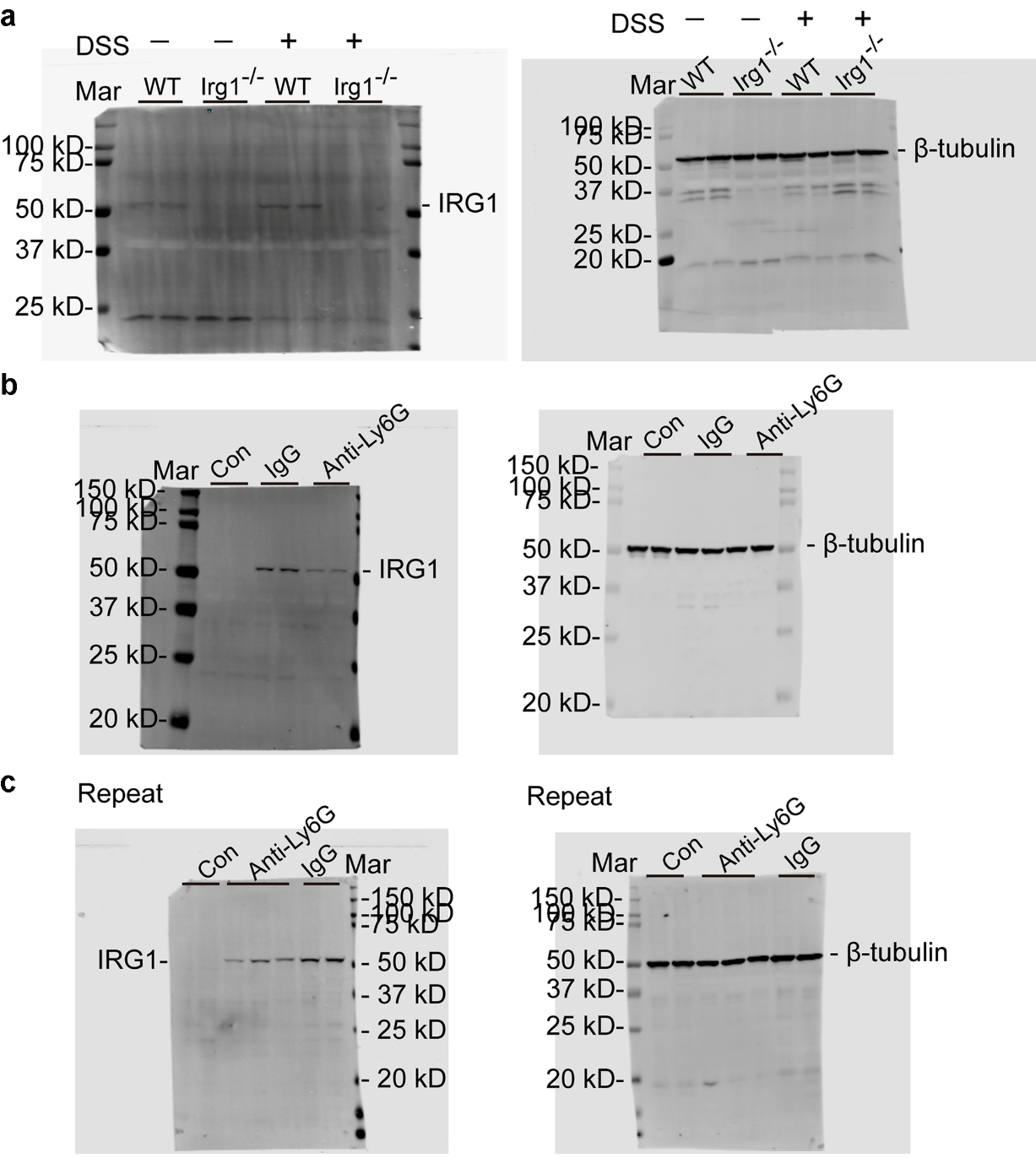


**a.** Original images corresponding to Supplementary Figure S3b. **b.** Original images corresponding to Figure 3g.**c.** Original images corresponding to Supplementary Figure S4g. IRG1 and β-tubulin (loading control) in colon tissue lysates. Molecular weight markers (kDa) are indicated on the left.
